# Delirium Post-Stroke: Short- and Long-Term Effect on Depression, Anxiety, Apathy and Aggression (Research Study—Part of PROPOLIS Study)

**DOI:** 10.3390/jcm9072232

**Published:** 2020-07-14

**Authors:** Katarzyna Kowalska, Jakub Droś, Małgorzata Mazurek, Paulina Pasińska, Agnieszka Gorzkowska, Aleksandra Klimkowicz-Mrowiec

**Affiliations:** 1 Department of Neurology, Faculty of Medicine, Medical College, Jagiellonian University, 31-503 Kraków, Poland; katarzyna.olga.kowalska@gmail.com (K.K.); paulinapotoczek@gmail.com (P.P.); 2 Doctoral School in Medical and Health Sciences, Jagiellonian University Medical College, 31-008 Kraków, Poland; jakub.dros@gmail.com; 3 Faculty of Medicine, Medical College, Jagiellonian University, 31-503 Kraków, Poland; mazurekmalgo@gmail.com; 4 Department of Neurorehabilitation, Faculty of Medical Sciences in Katowice, Medical University of Silesia, 40-752 Katowice, Poland; agorzkowska@sum.edu.pl

**Keywords:** stroke, post-stroke delirium, depression, anxiety, apathy, aggression

## Abstract

Background: Stroke patients are particularly vulnerable to delirium episodes, but very little is known about its subsequent adverse mental health outcomes. The author’s objective was to explore the association between in-hospital delirium and depression, anxiety, anger and apathy after stroke. Methods: A total of 750 consecutive patients with acute stroke or transient ischemic attack, were screened for delirium during hospitalization. Patients underwent mental health evaluation in hospital, 3 and 12 months post-stroke; depression, apathy, anxiety and anger were the outcomes measured at all evaluation check points. Results: Delirium was an independent risk factor for depression (OR = 2.28, 95%CI 1.15–4.51, *p* = 0.017) and aggression (OR = 3.39, 95%CI 1.48–7.73, *p* = 0.004) at the hospital, for anxiety 3 months post-stroke (OR = 2.83, 95%CI 1.25–6.39, *p* = 0.012), and for apathy at the hospital (OR = 4.82, 95%CI 2.25–10.47, *p* < 0.001), after 3 (OR = 3.84, 95%CI 1.31–11.21, *p* = 0.014) and 12 months (OR = 4.95, 95%CI 1.68–14.54, *p* = 0.004) post stroke. Conclusions: The results of this study confirm, that mental health problems are very frequent complications of stroke. Delirium in the acute phase of stroke influences mental health of patients. This effect is especially significant in the first months post-stroke and vanishes with time, which suggests that in-hospital delirium might not be a damaging occurrence in most measures of mental health problems from a long-term perspective.

## 1. Introduction

Among different neurological conditions stroke is a major risk factor for development of delirium [1]. Its prevalence in the in-hospital population ranges from 10.2 [2] to 48% [1]. The evidence suggests that, although the overt symptoms of delirium may be short lived, there may be a lasting impact of delirium on the long-term prognosis. There has been an increasing interest in the effect of delirium on healthcare outcomes. Previous research has linked post-stroke delirium with increased length of hospital stay [3], dependence, mortality [4], and cognitive impairment [5].

It has been shown that a substantial number of stroke survivors develop long term mental health problems such as anxiety, anger, depression, fear and others that have a negative influence on recovery, limit social reintegration of the persons with stroke, reduce quality of life, and are a source of caregiver burnout [6]. Moreover, previous research has associated delirium with an increased risk of long term mental health problems in different in-hospital populations; however, the data are equivocal [7]. Little is known about the effects of in-hospital delirium on mental health outcomes among stroke survivors.

One can hypothesize that delirium in acute phase of stroke can impact the mental health problems like depression, anxiety, apathy and anger. Therefore, the author’s objective in this prospective study was to investigate the influence of in-hospital delirium on patients’ psychiatric conditions in short- and long-term time perspective following stroke.

## 2. Materials and Methods

This study was conducted as part of a larger study, known as the PROPOLIS study, which investigated prevalence, risk factors, short- and long-term prognosis among patients with post-stroke delirium. Testing took place in the stroke unit at the University Hospital and Outpatients Clinique at the Neurology Department, University Hospital, Krakow. All procedures performed in this study involving human participants were in accordance with the ethical standards of the institutional and national research committee and with the 1964 Helsinki declaration and its later amendments. Informed written consent was provided by each participant or a caregiver. The Local Bioethics Committee of Jagiellonian University approved the study (KBET/63/B/2014).

### 2.1. Population and Design

The 750 consecutive patients with stroke (ischemic/hemorrhagic) or transient ischemic attack admitted to the stroke unit at the University Hospital in Krakow meeting the inclusion criteria for this study (Patients > 18 years of age, admitted within 48 h from the first stroke symptoms, speaking Polish) were investigated for the presence and risk factors of delirium. Patients were screened for delirium every day from admission to the 7th day of hospitalization with the abbreviated version of Confusion Assessment Method (bCAM) or the Intensive Care Units version (CAM-ICU), specifically in patients with motor aphasia or those who could not communicate for other reasons [8,9]. Delirium symptoms’ severity was assessed using the Cognitive Test for Delirium [10].

A resident neurologist trained in delirium diagnosis was responsible for screening for delirium, and a trained psychologist was responsible for cognitive, behavior/emotional assessment. The senior neurologist/neuropsychologist was responsible for evaluating all data. The physicians rating the patients in the hospital did not change during the study.

Due to the abrupt and fluctuating course of delirium, a short questionnaire regarding each patients’ behavior and cognitive status was completed by ward nurses throughout the entire hospitalization, 24 h per day, then analyzed by raters, in order not to miss any possible fluctuations of attention and awareness.

The final diagnosis of delirium was based on both clinical observation and structural assessment. The diagnostic criteria for delirium were based on the DSM-5 classification [11]. For those patients who were not able to undergo formal cognitive evaluation, the diagnosis was based on clinical observation and DSM-5 criteria for delirium. The raters were responsible for consensus of the diagnosis, if there were doubts then, the final diagnosis relied upon the senior neurologist.

Data were collected regarding socio-demographic factors and clinical features of patients. The details of the procedures were described elsewhere [4]. The Cumulative Illness Rating Scale (CIRS) was used as a general indicator of health status [12]. On admission information were obtained from the spouse/caregiver regarding pre-stroke mental and behavioral functioning on Neuropsychiatric Inventory [13]. In order to diagnose patients with pre-stroke dementia, a Polish version of Informant Questionnaire on Cognitive Decline in the Elderly (IQCODE) was used [14].

For cognitive assessment, the Montreal Cognitive Assessment (MoCA) [15] and Frontal Assessment Battery [16] were performed between day 1–2 and on the 7th day after admission.

All patients had neuroimaging (CT/MRI) performed on admission. Furthermore, upon admission, patients were screened for the severity of clinical deficit, which was graded by the National Institutes of Health Stroke Scale (NIHSS) [17]. Disabilities prior to admission were assessed by the modified Rankin Scale (mRS) [18].

The primary endpoint of this study was the influence of in-hospital delirium on mental health problems after 3 and 12 months post-stroke. The secondary end point was to assess the prevalence of depression, apathy, anxiety and anger after 3 and 12 months post-stroke.

### 2.2. Outcome Assessment

We assessed the following outcome measures: presence of depression, apathy, anxiety and anger (aggression/ hostility) between 7–10 days after admission to the hospital and during a follow-up visit 3 and 12 months after the stroke. Patients who did not attend a follow-up visit were contacted by phone and the information was gathered. A neurologist and a psychologist, both uninvolved in the baseline assessment of patients, were responsible for data acquisition.

### 2.3. Outcome Methods

The presence of depressive symptoms was assessed by Patient Health Questionnaire (PHQ-9) [19]. These items are used to query symptoms present over the last 2 weeks using 4-point Likert scale with item scores ranging from 0 (symptoms not present) to 3 (symptoms present nearly every day). The score ranges from 0 (no depressive symptoms) to 27 (all symptoms occurring nearly every day) and can be used to determine depression severity (0–4 indicates no depression, 5–9 mild depression, 10 to 14 moderate depression, 15–19 moderately severe depression and 20–27 severe depression). This method shows good reliability, validity and clinical utility when used in stroke patients [20].

To evaluate post-stroke apathy (PSA) the Apathy Evaluation Scale-C (AES-C) [21] was used. AES is an 18-item questionnaire with a clinician rated version that was applied in this study. The questions address patient’s activities, interest in doing things, relationship with others and feelings over the past two to three weeks. Each item is rated on a 4-point Likert scale with item scoring ranging from 1 (not at all true) to 4 (very true). The total AES-C score range from 18 to 72, with higher scores indicating greater apathy. The AES has good reliability and validity and was frequently used in studies on post-stroke apathy [21]. Apathy was diagnosed with AES score of ≥37 points [22].

Anxiety was measured with Polish adaptation of State Trait Anxiety Inventory (STAI) [23,24], the 40-item instrument, measuring respectively transient and enduring levels of anxiety. The state scale used in the present study administered as a self-completion questionnaire by the interviewer, assessed how the patients felt at the moment or in the recent past and how they anticipate their feelings to be in a specific, hypothetic situation in the future. STAI scale is scored on four levels of anxiety intensity from 1 (not at all) to 4 (very much) and with a sum score between 20 and 80. The raw results are interpreted by referring to a relevant sten scores and then categorized into three levels of anxiety: low (1–4 sten), moderate (5–6 sten) and high (7–10 sten) [24].

To assess anger, a Polish version of the Buss–Durkee Hostility Inventory (BDHI) was applied [25,26]. The BDHI is a 75-item questionnaire developed to assess Assault—physical violence against others; Indirect hostility—both roundabout and undirected aggression; Irritability—readiness to explode with negative affect at the slightest provocation; Negativism—oppositional behavior, usually against authority; Resentment—jealousy and hatred of others; Suspicion—projection of hostility onto others; Verbal hostility—negative affect expressed in both the style and content of speech; Guilt—feelings of being bad, having done wrong. The first seven sub-classes can be grouped into two factors. Resentment and Suspicion make up the factor Hostility, while Assault, Indirect hostility, Irritability and Verbal hostility sub-classes form the factor Aggression. The first factor, Hostility, reflects the cognitive components of anger while the Aggression factor reflects the behavioral components [26].

### 2.4. Statistics

Statistical analysis was performed using Statistica 13.3 software (StatSoft^®^, Kraków, Poland). Quantitative variables were presented as arithmetic means with standard deviations (SDs), or medians with interquartile ranges (IQRs), and depending on normal or non-normal distribution, were compared with the Student’s *t*-test or Mann–Whitney U test, respectively. Qualitative variables were compared using the chi-squared test with or without Yates’ correction. Considerable demographic and clinical factors were analyzed in univariate logistic regression models, and predictive values of delirium, presented as odds ratios (ORs) with 95% confidence intervals (CIs), on post-stroke depression, apathy, anxiety and aggression/hostility assessed in hospital and at follow-up visits were calculated. Then, delirium and other variables at *p*-value < 0.1 in the univariate analyses were included as potential predictors into multivariate logistic regression models in search of delirium as an independent risk factor using a forward stepwise selection method. *p-*values < 0.05 were considered statistically significant.

## 3. Results

**Figure 1:**
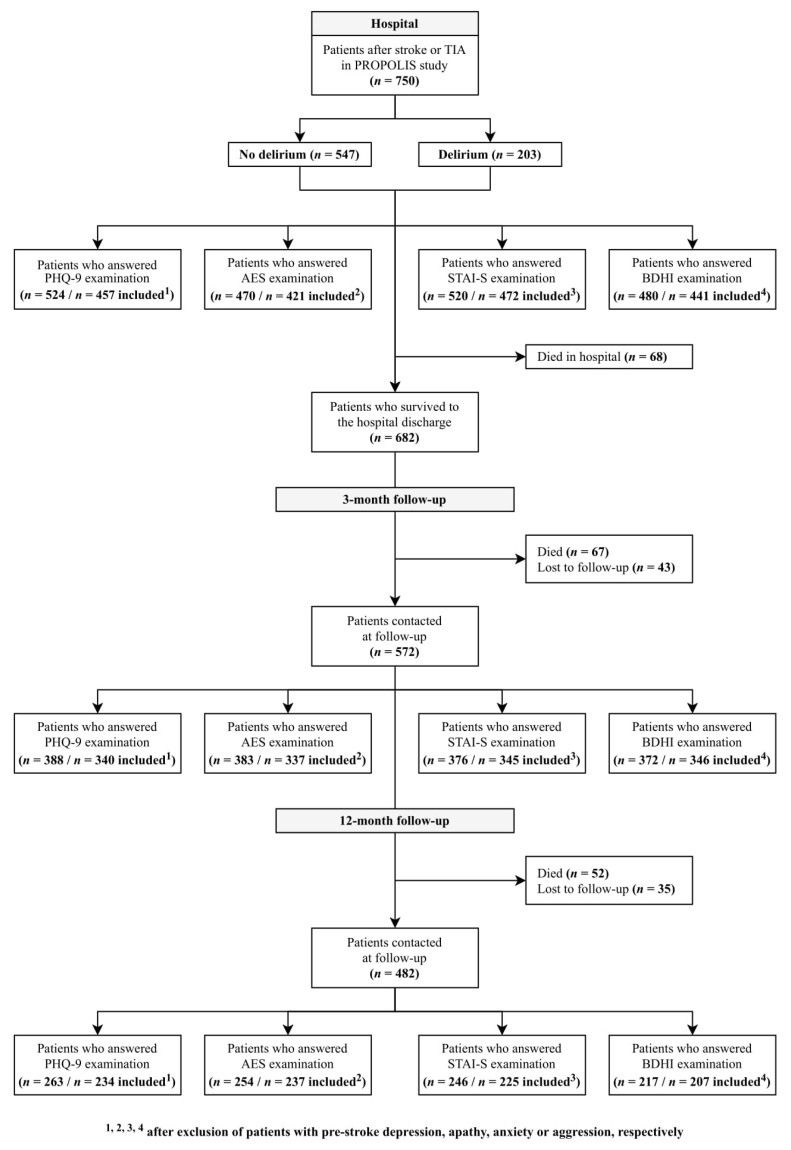
Study flowchart.

Of the 750 patients included to this study, 682 were dismissed from the hospital and scheduled for the follow-up visits. A flowchart shows the study design (Figure 1).

The prevalence of depression, apathy, fear and aggression/hostility at the hospital, 3 and 12 months post-stroke among patients with and without mental health problems prior to admission, among patients with and without delirium at the hospital is shown in Table 1 (the comparison of mental health problems’ prevalence between patients with and without delirium is shown in Table S1).

Patients with mental health problems prior to stroke were excluded from final analyses. The diagnosis of depression, anxiety, apathy and aggression was based on NPI on admission, the CIRS psychiatric/behavioral subscale, prior medical history, and medication actually taken by the patient.

In univariate analyses, delirium was a risk factor for depression, anxiety and hostility at the hospital and after 3 months, for aggression during hospitalization and for apathy during all of the observation period (see Supplementary Materials: Tables S2–S5). In multivariable logistic regression analysis, delirium was an independent risk factor for depression and aggression during hospitalization, anxiety after 3 months and apathy during all follow-up periods. Table 2 shows the final results.

The detailed results of all outcome measures during the follow-up period are available in Supplementary Data (Table S6).

Forty-three patients did not follow-up after 3 months and 35 after 12 months post-stroke. Those who were omitted after 3 months were significantly older, more physically and cognitively disabled prior to stroke. The patients that failed to follow-up 12 months after stroke were significantly older, had more severe neurological deficit on admission, and were more disabled prior to stroke. Tables S13 and S14 show the results.

## 4. Discussion

### 4.1. Brief Summary of the Findings

Despite the significant impact of in-hospital delirium on prognosis and long-term functional outcome for patients with stroke, its association with mental-health problems has not yet been clarified. Our study aimed to provide insight into the impact of delirium in acute phase of stroke on mental health in short and long time perspective while controlling for other relevant predisposing factors such as age, gender cognitive and vascular comorbidity.

### 4.2. Prevalence of Mental Health Problems Post-Stroke

In the meta-analysis by Hackett and Pickles [27], the pooled data showed that depression was present in 31% of stroke survivors at any time up to five-years post stroke; however, its frequency varied across studies from 5% at two to five days after stroke to 84% at 3-months after stroke. The rate of post-stroke depression was much higher in our study than average in meta-analysis. We observed this rate was very stable over the follow-up period.

The frequency of apathy following stroke is estimated between 20–25% [28]. In our study, the prevalence was higher, with a tendency to decrease its rate over 12 months post-stroke.

The prevalence of anxiety after the stroke in our study was similar to what was previously observed, both in the acute phase of stroke [29] as well as over the 12 months post-stroke. We also observed that the anxiety prevalence decreased with time.

Studies of anger after stroke are difficult to compare due to large differences. The reasons for this are the different time periods covered by each study, different methodologies, or different diagnostic criteria. A meta-analysis of 18 studies (5 in acute and 13 in post-acute time of stroke), showed that anger was diagnosed between 12 to 56% of patients [30]. We observed that behavioral and cognitive components of anger are not concurrent; aggression, behavior component, was low, less than 10%, in the acute phase of stroke and hostility, cognitive component, was high, close to 50%, among patients. We also observed its decreasing prevalence over 12 months post-stroke.

### 4.3. In-Hospital Delirium and Mental Health Problems

Delirium and depression, complex neuropsychiatric syndromes are common post stroke, however their relationship is vague. Still there is no clear answer to the question whether depressive symptoms arise during a delirium episode and if they resolve with delirium. The widespread disruption of neural networks in delirium may incorporate affective centers in vulnerable or predisposed subjects. Nelson et al. in the review article summarized that correlation exists between depression and delirium in patients with hip fracture, but in no other specific populations [31].

One small study, recently published, analyzed the short and long-term effect of delirium on anxiety and depression among stroke patients [32]. There was no influence of delirium on depression and anxiety 1-, 6- and 12-month post-stroke. In our study, the rate of depression was significantly higher in patients with delirium only 7–10 days after stroke and specifically when patients were analyzed regardless of dementia severity. It is unclear whether the depression symptoms measured soon after stroke truly represent new psychopathology or rather denote persistent or resolving delirium. Although studies have shown that depression is a risk factor for delirium [33,34,35], this relationship between delirium and depression is not clearly bidirectional and delirium seems to not increase the risk for depression in the long-term perspective.

In our study in-hospital delirium was an independent risk factor for apathy during all follow-up time. The association between apathy and delirium was not an area of interest for research so far, but these two clinical mental health problems have a common relationship. Apathy and delirium share a similar clinical picture. Delirium may manifest as lack of interest in an environment and goal-directed behavior, flat affect and withdrawal which resembles apathy [36].

Apathy and delirium share also similar anatomical correlations. Apathy is associated with disruption of medial frontal cortex—in particular the anterior corpus callosum, orbito-frontal cortex and subcortical structures including the ventral striatum, medial thalamus and ventral tegmental area, or connections between these regions. These associations are demonstrated across techniques that measure underlying neuronal metabolism, gray matter atrophy, and both structural and functional connectivity in the brain [37]. Delirium is associated with abnormalities of corpus callosum, cingulum, basal forebrain, occipital, parietal and temporal lobes, cerebellum and thalamus on diffusor tension imaging [38].

Presumably, silent structural brain lesions in critical regions for apathy and delirium cause higher brain vulnerability and as a result of stroke, clinically manifest as delirium or apathy or both.

In this study, we did not correlate the precise stroke lesion with mental health problem. However, studies show, that irrespective of the stroke lesion, patients with apathy have reduced connectivity in many regions, remote from stroke, including frontal and basal ganglia regions associated with apathy [39] as well as the reduced cerebral blood flow within the basal ganglia compared to non-apathetic patients, irrespective of stroke location [40].

Anger is a frequent complication of stroke. So far, no study reported the association between anger and delirium post-stroke. We observed such a relationship only in behavioral aspect of anger (aggression) during hospitalization. At that time, there was no relationship between cognitive component (hostility) of anger and delirium. We also did not find a positive association between delirium and aggression/hostility 3- and 12-month post-stroke. Activation studies have demonstrated that anger is associated with increased cerebral blood flow in brain regions that also show increased metabolism during delirium episode [41,42]. This could explain the association between aggression and delirium only during the early phase post-stroke.

Anxiety is a normal emotion to stress like stroke, hospitalization, fear of dying, but it may become persistent and inappropriate. Anxiety at the hospital was high in both groups, insignificantly higher in patients with delirium. After 3 months, delirium was found to be an independent risk factor for anxiety. Presumably, high emotional distress and memories of frightening psychotic experiences in delirious patients may be responsible for significantly higher rates of anxiety 3 months post-stroke when compare to patients without delirium. With time, when the memories faded, the rate of anxiety went down and its prevalence was similar in both groups.

This study confirms previous results, which show that mental health problems are a frequent post-stroke complication. This study builds upon previous research in that, for the first time, a large sample of stroke patients was assessed for the impact of in-hospital delirium on stroke patients’ mental health. Delirium is very rarely considered as a risk factor of long-term post-stroke prognosis. In our study, we evaluated disorders that are highly interrelated, such as delirium, depression, apathy, anxiety, and dementia. Assessments conducted at the same time are important to elucidate risk factors better, as well as the common and different mechanisms underlying those conditions.

### 4.4. Strengths and Weaknesses of the Study

The first step in arriving at a correct diagnosis of mental health problems is to distinguish delirium from other psychiatric syndromes that can cause confusion, such as dementia, depression, and mania. Evaluating different mental problems concurrently is also important, given the overlap between them. The careful and broad evaluation of mental health symptoms in stroke is a strong side of this study.

In our study, patients with aphasia, a frequent stroke consequence, were not excluded. If it was possible, CAM-ICU was administered; if patients could not be formally tested, then they were strictly observed by ward nurses and raters and the consensus diagnosis was made.

Since it is unclear to what degree previous psychopathology may serve as a risk factor for the development of mental health-problems after in-hospital delirium episode; prior psychiatric illness can influence mental status post-stroke, i.e., represents either recurrence or continuation of a preexisting psychiatric illness, we very carefully excluded patients with pre-morbid mental health problems.

This study included a big number of patients at the baseline that allowed a sustained reasonable big number of patients during all follow-ups.

A variety of raters; neurologist and psychologist assessed patients at baseline and during follow-up visits. This is considered as the strength of this study, because follow-up raters were blind for the patients’ previous performance and behavior. On the other hand, patients who are more familiar to assessors are more willing to ask for help if they have problems with understanding the questions from the questionnaire, and therefore provide more adequate answers. Therefore, the variety of raters can be also considered as a weakness of the study.

Some limitations of our study and bias inducers should also be addressed. Firstly, we used questionnaires to describe symptoms of anxiety, depression, apathy and fear that are not diagnostic tools, as using interviews with a mental health professional was not feasible. Secondly, patients that were lost in the follow-up had more risk factors, which could have had a negative influence on the outcome and been a potential source of bias. Thirdly, as this was a single center study, the generalizability of our results may be limited.

## 5. Conclusions

The results of this study show, that mental health problems are a very frequent post-stroke complication. Delirium diagnosed in the acute phase of stroke influences the mental health of affected patients. This effect is especially significant in the first months post-stroke and vanishes with time, which suggests that delirium during hospitalization might not be a damaging occurrence in most measures of mental health problems. Apathy seems to be the most connected mental health complication with in-hospital delirium among stroke patients.

## Figures and Tables

**Table 1:** The prevalence of depression, apathy, fear and aggression/hostility among patients with and without mental health problems prior to admission.

Time of Examination	Mental Health Problem	All Patients, *n* (%)	Patients without Delirium, *n* (%)	Patients with Delirium, *n* (%)
	**Depression ** PHQ-9 score ≥ 5			
Hospital		286/524 (54.58)	220/433 (58.81)	66/91 (72.53)
Hospital *		234/457 (51.20)	181/384 (47.14)	53/73 (72.60)
3 months		227/388 (58.51)	181/329 (55.02)	46/59 (77.97)
3 months *		191/340 (56.18)	157/294 (53.40)	34/46 (73.91)
12 months		144/263 (54.75)	127/234 (54.27)	17/29 (58.62)
12 months *		122/234 (52.14)	111/212 (52.36)	11/22 (50.00)
	**Apathy ** AES-C score ≥ 37			
Hospital		169/470 (35.96)	116/395 (39.27)	53/75 (70.67)
Hospital *		132/421 (31.35)	92/360 (25.56)	40/61 (65.57)
3 months		150/383 (39.16)	107/324 (33.02)	43/59 (72.88)
3 months *		117/337 (34.72)	89/294 (30.27)	28/43 (65.12)
12 months		70/254 (27.56)	53/226 (23.45)	17/28 (60.71)
12 months *		57/231 (24.68)	44/209 (21.05)	13/22 (59.09)
	**Anxiety ** STAI sten score ≥ 7			
Hospital		164/520 (31.54)	126/429 (29.37)	38/91 (41.76)
Hospital *		137/472 (29.03)	103/388 (26.55)	34/84 (40.48)
3 months		103/376 (27.39)	76/321 (23.68)	27/55 (49.09)
3 months *		93/345 (26.96)	68/294 (23.13)	35/51 (49.02)
12 months		48/246 (19.51)	41/219 (18.72)	7/27 (25.93)
12 months *		38/225 (16.89)	31/199 (15.58)	7/26 (26.92)
	**Aggression/hostility** BDHI score			
Hospital	‘Aggression’ sten score ≥ 7	45/480 (9.38)	32/401 (7.98)	13/79 (16.46)
	‘Hostility’ sten score ≥ 7	238/480 (49.58)	178/401 (44.39)	60/79 (75.95)
Hospital *		38/441 (8.62)	27/368 (7.34)	11/73 (15.07)
		213/441 (48.30)	158/368 (42.93)	55/73 (75.34)
3 months	‘Aggression’ sten score ≥ 7	37/372 (9.95)	31/318 (9.75%)	6/54 (11.11)
	‘Hostility’ sten score ≥ 7	79/372 (21.24)	63/318 (19.81)	16/54 (29.63)
3 months *		35/346 (10.12)	29/297 (9.76)	6/49 (12.24)
		74/346 (21.39)	58/297 (19.53)	16/49 (32.65)
12 months	‘Aggression’ sten score ≥ 7	16/217 (7.37)	14/197 (7.11)	2/20 (10.00)
	‘Hostility’ sten score ≥ 7	48/317 (22.12)	44/197 (22.34)	4/20 (20.00)
12 months *		15/207 (7.25)	13/187 (6.95)	2/20 (10.00)
		44/207 (21.26)	40/187 (21.39)	4/20 (20.00)

* number of patients with mental health problems after exclusion patients diagnosed with particular disorder prior to stroke; PHQ-9—Patient Health Questionnaire-9; AES—Apathy Evaluation Scale; STAI-S—State-Trait Anxiety Inventory, state scale; BDHI—Buss–Durkee Hostility Inventory; ‘Aggression’—assault, indirect hostility, irritability and verbal hostility; ‘Hostility’—resentment and suspicion

**Table 2:** Influence of post-stroke delirium on mental health problems during the follow-up period in univariate and multivariate logistic regression models.

	Mental Health Problem		Univariate Logistic Regression Model		Multivariate Logistic Regression Model	
Variable	No Delirium, *n* (%)	Delirium, *n* (%)	OR (95%CI)	*p*-Value	OR (95%CI)	*p*-Value
**Depression**						
*in hospital* PHQ-9 score ≥ 5	181/384 (47.14)	53/73 (72.60)	2.972 (1.711–5.162)	<0.001	2.286 (1.158–4.513)	0.017
**Apathy**						
*in hospital* AES score ≥ 37	92/360 (25.56)	40/61 (65.57)	5.549 (3.110–9.899)	<0.001	4.828 (2.225–10.477)	<0.001
*at 3-month follow-up* AES score ≥ 37	89/294 (30.20)	28/43 (65.12)	4.300 (2.190–8.442)	<0.001	3.841 (1.315–11.216)	0.014
*at 12-month follow-up* AES score ≥ 37	44/209 (21.05)	13/22 (59.09)	5.417 (2.175–13.493)	<0.001	4.951 (1.685–14.547)	0.004
**Anxiety**						
*at 3-month follow-up* STAI-S sten score ≥ 7	68/294 (23.13)	35/51 (49.02)	3.196 (1.732–5.895)	<0.001	2.831 (1.254–6.391)	0.012
**Aggression**						
*in hospital* BDHI ‘Aggression’ sten score ≥ 7	27/368 (7.34)	11/73 (15.07)	2.241 (1.057–4.751)	0.035	3.391 (1.486–7.739)	0.004

PHQ-9—Patient Health Questionnaire-9; AES—Apathy Evaluation Scale; STAI-S—State-Trait Anxiety Inventory, state scale; BDHI—Buss–Durkee Hostility Inventory; ‘Aggression’—assault, indirect hostility, irritability and verbal hostility.

## Supplementary Materials

The following are available online at https://www.mdpi.com/2077-0383/9/7/2232/s1, Table S1: Prevalence of depression, apathy, anxiety and aggression/hostility in hospital, at 3-month follow-up and 12-month follow-up in no-delirium and delirium groups, Table S2: Influence of post-stroke delirium on the incidence of depression in hospital, at 3-month follow-up and 12-month follow-up in univariate and multivariate logistic regression models, Table S3: Influence of post-stroke delirium on the incidence of apathy in hospital, at 3-month follow-up and 12-month follow-up in univariate and multivariate logistic regression models, Table S4: Influence of post-stroke delirium on the incidence of anxiety in hospital, at 3-month follow-up and 12-month follow-up in univariate and multivariate logistic regression models, Table S5: Influence of post-stroke delirium on the incidence of aggression/hostility in hospital, at 3-month follow-up and 12-month follow-up in univariate and multivariate logistic regression models, Table S6: Influence of post-stroke delirium on the incidence of depression, apathy, anxiety and aggression/hostility in hospital, at 3-month follow-up and 12-month follow-up in univariate and multivariate logistic regression models.
