# Supplementary material for: Delirium Post-Stroke: Short- and Long-Term Effect on Depression, Anxiety, Apathy and Aggression (Research Study—Part of PROPOLIS Study)
Source: J Clin Med. 2020 Jul 14;9(7):2232. doi: 10.3390/jcm9072232 (PMC7408940; doi:10.3390/jcm9072232)
Supplement: Supplementary file 1 [file jcm-09-02232-s001.pdf]

Table S1. Prevalence of depression, apathy, anxiety and aggression/hostility in hospital, at 3-month follow-up and 12-month follow-up in no-delirium and delirium groups

Variable	No delirium		Delirium		P-value
	Data	Score *	Data	Score *	
**Depression**					
PHQ-9 in hospital	384	4 (2-8)	73	7 (4-10)	**0.002**
PHQ-9 at 3m	294	5 (2-9)	46	8 (4-12)	**0.006**
PHQ-9 at 12m	212	5 (2-8)	22	4.5 (2-9)	0.771
**Apathy**					
AES in hospital	360	28 (21-37)	61	39 (34-47)	**<0.001**
AES at 3m	294	28 (22-40)	43	45 (32-57)	**<0.001**
AES at 12m	209	27 (22-35)	22	41.5 (27-55)	**<0.001**
**Anxiety**					
STAI-S in hospital	388	35 (28-44)	84	40.5 (31-48.5)	**0.003**
STAI-S at 3m	294	34 (26-43)	51	43 (31-50)	**0.002**
STAI-S at 12m	199	30 (26-37)	26	35 (39-44)	0.052
**Aggression/hostility**					
BDHI in hospital	368	48 (36-68)	73	60 (46-80)	**<0.001**
‘Aggression’ in hospital	368	30 (22-42)	73	36 (28-48)	**0.001**
‘Hostility’ in hospital	368	14 (8-22)	73	20 (16-26)	**<0.001**
Assault in hospital	368	6 (2-8)	73	8 (4-12)	**0.002**
Indirect hostility in hospital	368	6 (4-10)	73	8 (4-12)	0.954
Irritability in hospital	368	6 (4-10)	73	10 (6-10)	**<0.001**
Negativism in hospital	368	4 (2-6)	73	6 (2-8)	**<0.001**
Resentment in hospital	368	4 (2-8)	73	8 (6-12)	**<0.001**
Suspicion in hospital	368	10 (6-14)	73	12 (8-14)	**0.001**
Verbal hostility in hospital	368	12 (8-16)	73	14 (10-18)	**0.008**
Guilt in hospital	368	8.5 (6-14)	73	14 (10-16)	**<0.001**
BDHI at 3m	297	42 (30-57)	49	48 (26-72)	0.244
‘Aggression’ at 3m	297	29 (20-38)	49	32 (18-42)	0.474
‘Hostility’ at 3m	297	10 (6-15)	49	12 (7-19)	0.076
Assault at 3m	297	4 (2-7)	49	4 (2-8)	0.642
Indirect hostility at 3m	297	6 (4-10)	49	7 (6-10)	0.729
Irritability at 3m	297	8 (4-11)	49	10 (4-14)	0.052
Negativism at 3m	297	2 (0-4)	49	2 (0-6)	0.741
Resentment at 3m	297	4 (2-8)	49	6 (4-8)	**0.012**
Suspicion at 3m	297	5 (3-10)	49	6 (4-10)	0.329
Verbal hostility at 3m	297	10 (6-14)	49	8 (4-16)	0.546
Guilt at 3m	297	8 (5-10)	49	9 (6-12)	0.220
BDHI at 12m	187	44 (32-58)	20	38 (27-63.5)	0.635
‘Aggression’ at 12m	187	29 (20-39)	20	21.5 (14.5-37)	0.322
‘Hostility’ at 12m	187	12 (6-16)	20	10.5 (9.5-16)	0.416
Assault at 12m	187	4 (2-6)	20	3 (2-7)	0.987
Indirect hostility at 12m	187	6 (4-9)	20	4 (2-8)	0.119
Irritability at 12m	187	8 (4-11)	20	8 (6-13)	0.128
Negativism at 12m	187	3 (2-4)	20	2 (0-4)	0.299
Resentment at 12m	187	4 (2-8)	20	8 (4-10)	**0.021**
Suspicion at 12m	187	6 (4-10)	20	5.5 (2.5-9)	0.457
Verbal hostility at 12m	187	10 (6-15)	20	7.5 (4-13)	0.101
Guilt at 12m	187	8 (6-10)	20	7 (5.5-9.5)	0.608

* presented as median (IQR)

PHQ-9 – Patient Health Questionnaire-9; AES – Apathy Evaluation Scale; STAI-S – State-Trait Anxiety Inventory, state scale; BDHI – Buss-Durkee Hostility Inventory; ‘Aggression’ – assault, indirect hostility, irritability and verbal hostility; ‘Hostility’ – resentment and suspicion; at 3m – at 3-month follow-up; at 12m – at 12-month follow-up

Table S2. Influence of post-stroke delirium on the incidence of depression in hospital, at 3-month follow-up and 12-month follow-up in univariate and multivariate logistic regression models

	Incidence * of depression		Univariate logistic regression model			Multivariate logistic regression model		
**Variable**	**No delirium**	**Delirium**	**OR**	**95%CI**	**P-value**	**OR**	**95%CI**	**P-value**
In hospital								
PHQ-9 score ≥5	181/384 (47.14%)	53/73 (72.60%)	**2.972**	**1.711-5.162**	**<0.001**	**2.286**	**1.158-4.513**	**0.017**
PHQ-9 score ≥10	80/384 (20.83%)	20/73 (27.40%)	1.434	0.811-2.536	0.215	ns	ns	ns
PHQ-9 score ≥15	24/384 (6.25%)	1/73 (1.37%)	0.208	0.028-1.565	0.127	ns	ns	ns
PHQ-9 score ≥20	3/384 (0.78%)	0/73 (0%)	-	-	-	-	-	-
At 3-motnh follow-up								
PHQ-9 score ≥5	157/294 (53.40%)	34/46 (73.91%)	**2.472**	**1.232-4.963**	**0.011**	ns	ns	ns
PHQ-9 score ≥10	70/294 (23.81%)	19/46 (41.30%)	**2.252**	**1.181-4.294**	**0.014**	ns	ns	ns
PHQ-9 score ≥15	29/294 (9.86%)	9/46 (19.57%)	2.223	0.976-5.063	0.057	ns	ns	ns
PHQ-9 score ≥20	6/294 (2.04%)	3/46 (6.52%)	3.349	0.807-13.889	0.096	ns	ns	ns
At 12-month follow-up								
PHQ-9 score ≥5	111/212 (52.36%)	11/22 (50.00%)	0.910	0.378-2.190	0.833	ns	ns	ns
PHQ-9 score ≥10	43/212 (20.28%)	5/22 (22.73%)	1.156	0.404-3.309	0.787	ns	ns	ns
PHQ-9 score ≥15	9/212 (4.25%)	3/22 (13.64%)	3.561	0.888-14.280	0.073	ns	ns	ns
PHQ-9 score ≥20	1/212 (0.47%)	0/22 (0%)	-	-	-	-	-	-

* presented as n (%)

PHQ-9 – Patient Health Questionnaire-9; ns – not significant

Table S3. Influence of post-stroke delirium on the incidence of apathy in hospital, at 3-month follow-up and 12-month follow-up in univariate and multivariate logistic regression models

	Incidence * of apathy		Univariate logistic regression model			Multivariate logistic regression model		
**Variable**	**No delirium**	**Delirium**	**OR**	**95%CI**	**P-value**	**OR**	**95%CI**	**P-value**
In hospital								
AES score ≥37	92/360 (25.56%)	40/61 (65.57%)	**5.549**	**3.110-9.899**	**<0.001**	**4.828**	**2.225-10.477**	**<0.001**
At 3-motnh follow-up								
AES score ≥37	89/294 (30.27%)	28/43 (65.12%)	**4.300**	**2.190-8.442**	**<0.001**	**3.841**	**1.315-11.216**	**0.014**
At 12-month follow-up								
AES score ≥37	44/209 (21.05%)	13/22 (59.09%)	**5.417**	**2.175-13.493**	**<0.001**	**4.951**	**1.685-14.547**	**0.004**

* presented as n (%)
AES – Apathy Evaluation Scale

Table S4. Influence of post-stroke delirium on the incidence of anxiety in hospital, at 3-month follow-up and 12-month follow-up in univariate and multivariate logistic regression models

	Incidence * of anxiety		Univariate logistic regression model			Multivariate logistic regression model		
**Variable**	**No delirium**	**Delirium**	**OR**	**95%CI**	**P-value**	**OR**	**95%CI**	**P-value**
In hospital								
STAI-S sten score of ≥7	103/388 (26.55%)	34/84 (40.48%)	**1.882**	**1.152-3.073**	**0.012**	ns	ns	ns
At 3-motnh follow-up								
STAI-S sten score of ≥7	68/294 (23.13%)	35/51 (49.02%)	**3.196**	**1.732-5.895**	**<0.001**	**2.831**	**1.254-6.391**	**0.012**
At 12-month follow-up								
STAI-S sten score of ≥7	31/199 (15.58%)	7/26 (26.92%)	1.997	0.774-5.150	0.153	ns	ns	ns

* presented as n (%)

STAI-S – State-Trait Anxiety Inventory, state scale; ns – not significant

Table S5. Influence of post-stroke delirium on the incidence of aggression/hostility in hospital, at 3-month follow-up and 12-month follow-up in univariate and multivariate logistic regression models

	Incidence * of aggression/hostility		Univariate logistic regression model			Multivariate logistic regression model		
**Variable**	**No delirium**	**Delirium**	**OR**	**95%CI**	**P-value**	**OR**	**95%CI**	**P-value**
In hospital								
'Aggression' sten score of ≥7	27/368 (7.34%)	11/73 (15.07%)	**2.241**	**1.057-4.751**	**0.035**	**3.391**	**1.486-7.739**	**0.004**
'Hostility' sten score of ≥7	158/368 (42.93%)	55/73 (75.34%)	**4.061**	**2.295-7.187**	**<0.001**	ns	ns	ns
Assault sten score of ≥7	79/368 (21.47%)	35/73 (47.95%)	**3.369**	**1.999-5.680**	**<0.001**	**2.545**	**1.176-5.508**	**0.018**
Indirect hostility sten score of ≥7	105/368 (28.53%)	21/73 (28.77%)	1.012	0.581-1.762	0.968	ns	ns	ns
Irritability sten score of ≥7	14/368 (3.80%)	1/73 (1.37%)	0.351	0.045-2.713	0.316	ns	ns	ns
Negativism sten score of ≥7	135/368 (36.68%)	42/73 (57.53%)	**2.338**	**1.404-3.895**	**0.001**	**1.861**	**1.067-3.244**	**0.029**
Resentment sten score of ≥7	124/368 (33.70%)	45/73 (61.64%)	**3.162**	**1.882-5.314**	**<0.001**	ns	ns	ns
Suspicion sten score of ≥7	219/368 (59.51%)	58/73 (79.45%)	**2.631**	**1.437-4.816**	**0.002**	ns	ns	ns
Verbal hostility sten score of ≥7	80/368 (21.74%)	30/73 (41.10%)	**2.512**	**1.481-4.258**	**<0.001**	**2.774**	**1.518-5.068**	**0.001**
Guilt sten score of ≥7	139/368 (37.77%)	44/73 (60.27%)	**2.500**	**1.495-4.179**	**<0.001**	**1.876**	**1.024-3.438**	**0.042**
At 3-motnh follow-up								
'Aggression' sten score of ≥7	29/297 (9.76%)	6/49 (12.24%)	1.289	0.506-3288	0.595	ns	ns	ns
'Hostility' sten score of ≥7	58/297 (19.53%)	16/49 (32.65%)	**1.998**	**1.030-3.875**	**0.041**	ns	ns	ns
Assault sten score of ≥7	35/297 (11.78%)	9/49 (18.37%)	1.684	0.753-3.766	0.204	ns	ns	ns
Indirect hostility sten score of ≥7	98/297 (33.00%)	17/49 (34.69%)	1.079	0.571-2.038	0.815	ns	ns	ns
Irritability sten score of ≥7	26/297 (8.75%)	8/49 (16.33%)	2.034	0.863-4.796	0.105	ns	ns	ns
Negativism sten score of ≥7	67/297 (22.56%)	14/49 (28.57%)	1.373	0.698-2.702	0.359	ns	ns	ns
Resentment sten score of ≥7	83/297 (27.95%)	18/49 (36.73%)	1.497	0.794-2.821	0.212	ns	ns	ns
Suspicion sten score of ≥7	92/297 (30.98%)	17/49 (34.69%)	1.184	0.626-2.240	0.604	ns	ns	ns
Verbal hostility sten score of ≥7	47/297 (15.82%)	11/49 (22.45%)	1.540	0.735-3.227	0.253	ns	ns	ns
Guilt sten score of ≥7	69/297 (23.23%)	16/49 (32.65%)	1.602	0.832-3.084	0.158	ns	ns	ns
At 12-month follow-up								
'Aggression' sten score of ≥7	13/187 (6.95%)	2/20 (10.00%)	1.487	0.311-7.119	0.619	ns	ns	ns
'Hostility' sten score of ≥7	40/187 (21.39%)	4/20 (20.00%)	0.919	0.291-2.902	0.885	ns	ns	ns
Assault sten score of ≥7	17/187 (9.09%)	4/20 (20.00%)	2.500	0.750-8.332	0.136	ns	ns	ns
Indirect hostility sten score of ≥7	46/187 (24.60%)	3/20 (15.00%)	0.541	0.152-1.929	0.344	ns	ns	ns
Irritability sten score of ≥7	17/187 (9.09%)	4/20 (20.00%)	2.500	0.750-8.332	0.136	ns	ns	ns
Negativism sten score of ≥7	29/187 (20.86%)	3/20 (15.00%)	0.670	0.187-2.402	0.538	ns	ns	ns
Resentment sten score of ≥7	57/187 (30.48%)	12/20 (60.00%)	**3.421**	**1.327-8.822**	**0.011**	**3.857**	**1.314-11.322**	**0.014**
Suspicion sten score of ≥7	58/187 (31.02%)	5/20 (25.00%)	0.741	0.257-2.137	0.580	ns	ns	ns
Verbal hostility sten score of ≥7	35/187 (18.72%)	2/20 (10.00%)	0.483	0.107-2.176	0.343	ns	ns	ns
Guilt sten score of ≥7	36/187 (19.25%)	2/20 (10.00%)	0.466	0.103-2.100	0.320	ns	ns	ns

* presented as n (%)

'Aggression' – assault, indirect hostility, irritability and verbal hostility;

'Hostility' – resentment and suspicion; ns – not significant

Table S6. Influence of post-stroke delirium on the incidence of depression, apathy, anxiety and aggression/hostility in hospital, at 3-month follow-up and 12-month follow-up in univariate and multivariate logistic regression models

	Incidence *		Univariate logistic regression model			Multivariate logistic regression model		
**Variable**	**No delirium**	**Delirium**	**OR**	**95%CI**	**P-value**	**OR**	**95%CI**	**P-value**
**Depression**								
In hospital								
PHQ-9 score ≥5	181/384 (47.14%)	53/73 (72.60%)	**2.972**	**1.711-5.162**	**<0.001**	**2.286**	**1.158-4.513**	**0.017**
PHQ-9 score ≥10	80/384 (20.83%)	20/73 (27.40%)	1.434	0.811-2.536	0.215	ns	ns	ns
PHQ-9 score ≥15	24/384 (6.25%)	1/73 (1.37%)	0.208	0.028-1.565	0.127	ns	ns	ns
PHQ-9 score ≥20	3/384 (0.78%)	0/73 (0%)	-	-	-	-	-	-
At 3-motnh follow-up								
PHQ-9 score ≥5	157/294 (53.40%)	34/46 (73.91%)	**2.472**	**1.232-4.963**	**0.011**	ns	ns	ns
PHQ-9 score ≥10	70/294 (23.81%)	19/46 (41.30%)	**2.252**	**1.181-4.294**	**0.014**	ns	ns	ns
PHQ-9 score ≥15	29/294 (9.86%)	9/46 (19.57%)	2.223	0.976-5.063	0.057	ns	ns	ns
PHQ-9 score ≥20	6/294 (2.04%)	3/46 (6.52%)	3.349	0.807-13.889	0.096	ns	ns	ns
At 12-month follow-up								
PHQ-9 score ≥5	111/212 (52.36%)	11/22 (50.00%)	0.910	0.378-2.190	0.833	ns	ns	ns
PHQ-9 score ≥10	43/212 (20.28%)	5/22 (22.73%)	1.156	0.404-3.309	0.787	ns	ns	ns
PHQ-9 score ≥15	9/212 (4.25%)	3/22 (13.64%)	3.561	0.888-14.280	0.073	ns	ns	ns
PHQ-9 score ≥20	1/212 (0.47%)	0/22 (0%)	-	-	-	-	-	-
**Apathy**								
In hospital								
AES score ≥37	92/360 (25.56%)	40/61 (65.57%)	**5.549**	**3.110-9.899**	**<0.001**	**4.828**	**2.225-10.477**	**<0.001**
At 3-motnh follow-up								
AES score ≥37	89/294 (30.27%)	28/43 (65.12%)	**4.300**	**2.190-8.442**	**<0.001**	**3.841**	**1.315-11.216**	**0.014**
At 12-month follow-up								
AES score ≥37	44/209 (21.05%)	13/22 (59.09%)	**5.417**	**2.175-13.493**	**<0.001**	**4.951**	**1.685-14.547**	**0.004**
**Anxiety**								
In hospital								
STAI-S sten score of ≥7	103/388 (26.55%)	34/84 (40.48%)	**1.882**	**1.152-3.073**	**0.012**	ns	ns	ns
At 3-motnh follow-up								
STAI-S sten score of ≥7	68/294 (23.13%)	35/51 (49.02%)	**3.196**	**1.732-5.895**	**<0.001**	**2.831**	**1.254-6.391**	**0.012**
At 12-month follow-up								
STAI-S sten score of ≥7	31/199 (15.58%)	7/26 (26.92%)	1.997	0.774-5.150	0.153	ns	ns	ns
**Aggression/hostility**								
In hospital								
'Aggression' sten score of ≥7	27/368 (7.34%)	11/73 (15.07%)	**2.241**	**1.057-4.751**	**0.035**	**3.391**	**1.486-7.739**	**0.004**
'Hostility' sten score of ≥7	158/368 (42.93%)	55/73 (75.34%)	**4.061**	**2.295-7.187**	**<0.001**	ns	ns	ns
At 3-motnh follow-up								
'Aggression' sten score of ≥7	29/297 (9.76%)	6/49 (12.24%)	1.289	0.506-3288	0.595	ns	ns	ns
'Hostility' sten score of ≥7	58/297 (19.53%)	16/49 (32.65%)	**1.998**	**1.030-3.875**	**0.041**	ns	ns	ns
At 12-month follow-up								
'Aggression' sten score of ≥7	13/187 (6.95%)	2/20 (10.00%)	1.487	0.311-7.119	0.619	ns	ns	ns
'Hostility' sten score of ≥7	40/187 (21.39%)	4/20 (20.00%)	0.919	0.291-2.902	0.885	ns	ns	ns

* presented as n (%)

PHQ-9 – Patient Health Questionnaire-9; AES – Apathy Evaluation Scale; STAI-S – State-Trait Anxiety Inventory, state scale; 'Aggression' – assault, indirect hostility, irritability and verbal hostility; 'Hostility' – resentment and suspicion; ns – not significant

Table S7. Predictors of post-stroke depression (PHQ-9 score ≥5) in hospital in univariate and multivariate logistic regression models

				Univariate logistic regression model			Multivariate logistic regression model		
**Variable**	**Data**	**No depression**	**Depression**	**OR**	**95%CI**	**P-value**	**OR**	**95%CI**	**P-value**
Male gender *	457	139/223 (62.33%)	104/234 (44.44%)	**0.483**	**0.333-0.703**	**<0.001**	**0.400**	**0.249-0.642**	**<0.001**
Age [years] **	457	67 (30-78)	73 (62-80)	**1.019**	**1.005-1.034**	**0.008**	ns	ns	ns
BMI [kg/m^2] **	447	26.11 (23.64-29.68)	27.34 (24.06-30.47)	1.020	0.981-1.061	0.315	ns	ns	ns
Higher education *	451	47 (21.36%)	40/231 (17.32%)	0.771	0.482-1.232	0.280	ns	ns	ns
Education length [years] **	449	12 (10-14)	11 (9-13)	0.951	0.899-1.006	0.081	ns	ns	ns
Hemorrhagic stroke *	457	12/223 (5.38%)	12/234 (5.13%)	0.950	0.418-2.162	0.904	ns	ns	ns
TOAST classification									
- 1 *	398	24/197 (12.18%)	22/201 (10.95%)	0.886	0.479-1.639	0.700	ns	ns	ns
- 2 *	398	11/197 (5.58%)	11/201 (5.47%)	0.979	0.414-2.313	0.961	ns	ns	ns
- 3 *	398	59/197 (29.95%)	65/201 (32.34%)	1.118	0.731-1.709	0.607	ns	ns	ns
- 4 *	398	101 (51.27%)	101/201 (50.25%)	0.960	0.648-1.422	0.839	ns	ns	ns
- 5 *	398	2/197 (1.02%)	2/201 (1.00%)	0.980	0.137-7.026	0.984	ns	ns	ns
Side of stroke									
- right hemisphere *	457	88/223 (39.46%)	108/234 (46.15%)	1.315	0.907-1.907	0.149	ns	ns	ns
- left hemisphere *	457	102/223 (45.74%)	88/234 (37.61%)	0.715	0.492-1.039	0.078	ns	ns	ns
- posterior part *	457	31/223 (13.90%)	30/234 (12.82%)	0.911	0.531-1.562	0.734	ns	ns	ns
- multiple locations *	457	2/223 (0.90%)	8/23 (3.42%)	3.912	0.821-18.625	0.087	ns	ns	ns
rt-Pa treatment *	457	61/223 (27.35%)	41/234 (17.52%)	**0.564**	**0.361-0.883**	**0.012**	ns	ns	ns
Thrombectomy *	457	11/223 (4.93%)	9/234 (3.85%)	0.771	0.313-1.897	0.571	ns	ns	ns
Medical history									
- hypertension *	457	148/223 (66.37%)	172/234 (73.50%)	1.406	0.941-2.101	0.097	ns	ns	ns
- diabetes *	457	44/223 (19.73%)	76/234 (32.48%)	**1.957**	**1.275-3.004**	**0.002**	ns	ns	ns
- atrial fibrillation *	457	35/223 (15.70%)	44/234 (18.80%)	1.244	0.764-2.026	0.380	ns	ns	ns
- myocardial infraction *	457	32/223 (14.35%)	32/234 (13.68%)	0.946	0.557-1.604	0.836	ns	ns	ns
- PCI or CABG *	457	22/223 (9.87%)	20/234 (8.55%)	0.854	0.452-1.612	0.626	ns	ns	ns
- smoking – ever *	456	112/222 (50.45%)	118/234 (50.43%)	0.999	0.692-1.442	0.996	ns	ns	ns
- smoking – current *	456	59/222 (26.58%)	66/234 (28.21%)	1.085	0.719-1.639	0.697	ns	ns	ns
- previous stroke or TIA *	455	30/222 (13.51%)	49/233 (21.03%)	**1.704**	**1.037-2.803**	**0.036**	ns	ns	ns
CIRS, total score **	457	7 (4-11)	9 (6-12)	**1.097**	**1.051-1.146**	**<0.001**	ns	ns	ns
Medicines									
- anticholinergic risk scale ***	419	0.02 ± 0.22	0.11 ± 0.69	1.771	0.892-3.519	0.103	ns	ns	ns
- antidepressants *	375	0/188 (0%)	3/187 (1.60%)	-	-	-	-	-	-
- neuroleptics *	375	2/188 (1.06%)	1/187 (0.53%)	0.500	0.045-5.562	0.573	ns	ns	ns
- benzodiazepines *	374	3/187 (1.60%)	4/187 (2.14%)	1.341	0.296-6.074	0.704	ns	ns	ns
Pneumonia *	457	7/223 (3.14%)	21/234 (8.97%)	**3.042**	**1.267-7.306**	**0.013**	**3.360**	**1.005-11.237**	**0.049**
Urinary tract infections *	442	52/218 (23.85%)	68/224 (30.36%)	1.392	0.912-2.122	0.125	ns	ns	ns
Hospital stay [days] **	457	9 (8-10)	9 (8-11)	1.033	0.982-1.087	0.205	ns	ns	ns
Aphasia in hospital *	457	48/223 (21.52%)	40/234 (17.09%)	0.752	0.471-1.199	0.231	ns	ns	ns
Neglect in hospital *	457	20/223 (8.97%)	28/234 (11.97%)	1.380	0.752-2.528	0.298	ns	ns	ns
Vision deficits in hospital *	457	53/223 (23.77%)	71/234 (30.34%)	1.397	0.922-2.117	0.115	ns	ns	ns

NIHSS at admission **	457	4 (2-7)	4 (2-9)	1.008	0.974-1.044	0.641	ns	ns	ns
Pre-hospital mRS **	457	0 (0-0)	0 (0-1)	**1.488**	**1.189-1.863**	**<0.001**	**1.433**	**1.032-1.990**	**0.032**
CRP in hospital [mg/l] **	442	3.74 (1.59-10.77)	5.44 (1.97-17.25)	**1.007**	**1.001-1.013**	**0.030**	ns	ns	ns
NPI, total score ***	374	2.57 ± 5.49	5.92 ± 11.15	**1.061**	**1.025-1.099**	**<0.001**	**1.045**	**1.008-1.084**	**0.018**
- agitation/aggression ***	374	1.23 ± 3.30	2.01 ± 5.80	1.039	0.989-1.091	0.129	ns	ns	ns
- mood ***	374	0.93 ± 2.79	1.28 ± 2.81	1.048	0.971-1.130	0.228	ns	ns	ns
- frontal ***	374	1.07 ± 2.96	1.45 ± 3.47	1.038	0.972-1.108	0.264	ns	ns	ns
- psychosis ***	374	0.10 ± 0.60	0.59 ± 2.80	1.245	0.987-1.569	0.064	ns	ns	ns
Pre-hospital IQCODE **	377	78 (78-79)	78 (78-81)	**1.058**	**1.020-1.097**	**0.003**	ns	ns	ns
Delirium in hospital *	457	20/223 (8.97%)	53/234 (22.65%)	**2.972**	**1.711-5.162**	**<0.001**	**2.286**	**1.158-4.513**	**0.017**
Delirium type									
- hyperactive *	457	4/223 (1.79%)	13/234 (5.56%)	**3.221**	**1.034-10.031**	**0.044**	ns	ns	ns
- hypoactive *	457	5/223 (2.24%)	23/234 (9.83%)	**4.753**	**1.774-12.733**	**0.002**	ns	ns	ns
- mixed *	457	6/223 (2.69%)	14/234 (5.98%)	2.302	0.868-6.099	0.094	ns	ns	ns
Delirium length [days] **	73	3 (1-6.5)	2 (1-5)	0.920	0.736-1.149	0.461	ns	ns	ns

* n (%); ** median (IQR); *** mean ± SD; PHQ-9 – Patient Health Questionnaire-9; BMI – body mass index; TOAST – Trial of Org 10172 in Acute Stroke Treatment; rt-Pa – recombinant tissue plasminogen activator; PCI – percutaneous coronary interventions; CABG – coronary artery bypass graft; TIA – transient ischemic attack; CIRS – Cumulative Illness Rating Scale; NIHSS – National Institutes of Health Stroke Scale; mRS – Modified Rankin Scale; CRP – C-reactive protein; NPI – Neuropsychiatric Inventory; IQCODE - Informant Questionnaire on Cognitive Decline in the Elderly; ns – not significant

Table S8. Predictors of post-stroke apathy (AES score ≥ 37) in hospital in univariate and multivariate logistic regression models

				Univariate logistic regression model			Multivariate logistic regression model		
**Variable**	**Data**	**No apathy**	**Apathy**	**OR**	**95%CI**	**P-value**	**OR**	**95%CI**	**P-value**
Male gender *	421	142/289 (49.13%)	73/132 (55.30%)	1.281	0.847-1.937	0.241	ns	ns	ns
Age [years] **	421	66 (59-77)	74.5 (64.5-81)	**1.041**	**1.023-1.059**	**<0.001**	ns	ns	ns
BMI [kg/m ^{2}] **	413	26.54 (23.59-30.06)	27.34 (24.69-31.02)	1.032	0.990-1.076	0.135	ns	ns	ns
Higher education *	416	68/285 (23.86%)	14/131 (10.69%)	**0.382**	**0.206-0.708**	**0.002**	ns	ns	ns
Education length [years] **	414	7 (4-11)	10 (9-12)	**0.874**	**0.816-0.937**	**<0.001**	ns	ns	ns
Hemorrhagic stroke *	421	13/289 (4.50%)	9/132 (6.82%)	1.553	0.647-3.731	0.324	ns	ns	ns
TOAST classification									
- 1 *	371	23/257 (8.95%)	17/114 (14.91%)	1.783	0.912-3.485	0.091	ns	ns	ns
- 2 *	371	15/257 (5.84%)	2/114 (1.75%)	0.288	0.065-1.281	0.102	ns	ns	ns
- 3 *	371	70/257 (27.24%)	44/114 (38.60%)	**1.679**	**1.053-2.677**	**0.029**	ns	ns	ns
- 4 *	371	146/257 (56.81%)	50/114 (43.86%)	**0.594**	**0.381-0.927**	**0.022**	ns	ns	ns
- 5 *	371	3/257 (1.117%)	1/114 (0.88%)	0.749	0.077-7.282	0.804	ns	ns	ns
Side of stroke									
- right hemisphere *	421	125/289 (43.25%)	60/132 (45.45%)	1.093	0.723-1.654	0.673	ns	ns	ns
- left hemisphere *	421	117/289 (40.48%)	50/132 (37.88%)	0.896	0.587-1.368	0.612	ns	ns	ns
- posterior part *	421	41/289 (14.19%)	19/132 (14.39%)	1.017	0.565-1.831	0.955	ns	ns	ns
- multiple locations *	421	6/289 (2.08%)	3/132 (2.27%)	1.097	0.270-4.455	0.897	ns	ns	ns
rt-Pa treatment *	421	65/28 (22.49%)	34/132 (25.76%)	1.196	0.741-1.928	0.464	ns	ns	ns
Thrombectomy *	421	11/289 (3.81%)	7/132 (5.30%)	1.415	0.536-3.737	0.483	ns	ns	ns
Medical history									
- hypertension *	421	187/289 (64.71%)	98/132 (74.24%)	1.572	0.994-2.487	0.053	ns	ns	ns
- diabetes *	421	52/289 (17.99%)	54/132 (40.91%)	**3.155**	**1.994-4.992**	**<0.001**	**2.927**	**1.563-5.479**	**0.001**
- atrial fibrillation *	421	40/289 (13.84%)	34/132 (25.76%)	**2.160**	**1.292-3.609**	**0.003**	ns	ns	ns
- myocardial infraction *	421	31/289 (10.73%)	22/32 (16.67%)	1.665	0.922-3.003	0.091	ns	ns	ns
- PCI or CABG *	421	22/289 (7.61%)	14/132 (10.61%)	1.440	0.712-2.912	0.310	ns	ns	ns
- smoking – ever *	420	149/288 (51.74%)	69/132 (52.27%)	1.022	0.676-1.543	0.919	ns	ns	ns
- smoking – current *	420	79/288 (27.43%)	41/132 (31.06%)	1.192	0.760-1.870	0.445	ns	ns	ns
- previous stroke or TIA *	420	46/288 (15.97%)	26/132 (19.70%)	1.290	0.758-2.197	0.348	ns	ns	ns
CIRS, total score **	421	7 (4-11)	10 (6-12.5)	**1.099**	**1.049-1.150**	**<0.001**	ns	ns	ns
Medicines									
- anticholinergic risk scale ***	382	0.01 ± 0.18	0.22 ± 0.87	**2.806**	**1.244-6.330**	**0.013**	ns	ns	ns
- antidepressants *	353	3/245 (1.22%)	5/108 (4.63%)	3.916	0.919-16.691	0.065	ns	ns	ns
- neuroleptics *	353	2/245 (0.82%)	0/108 (0%)	-	-	-	-	-	-
- benzodiazepines *	352	4/244 (1.64%)	2/108 (1.85%)	1.132	0.204-6.276	0.887	ns	ns	ns
Pneumonia *	421	13/289 (4.50%)	9/132 (6.82%)	1.553	0.647-3.731	0.324	ns	ns	ns
Urinary tract infections *	409	69 (24.56%)	44/128 (34.38%)	**1.609**	**1.021-2.536**	**0.040**	ns	ns	ns
Hospital stay [days] **	421	9 (8-10)	9 (9-12.5)	**1.058**	**1.002-1.116**	**0.043**	ns	ns	ns
Aphasia in hospital *	421	50/289 (17.30%)	27/132 (20.45%)	1.229	0.730-2.070	0.438	ns	ns	ns
Neglect in hospital *	421	24/289 (8.30%)	19/132 (14.39%)	1.857	0.978-3.524	0.059	ns	ns	ns
Vision deficits in hospital *	421	60/289 (20.76%)	53/132 (40.15%)	**2.561**	**1.634-4.013**	**<0.001**	ns	ns	ns

NIHSS at admission **	421	4 (2-7)	5.5 (3-10)	**1.054**	**1.014-1.095**	**0.008**	ns	ns	ns
Pre-hospital mRS **	421	0 (0-0)	0 (0-1)	**1.435**	**1.160-1.775**	**<0.001**	ns	ns	ns
CRP in hospital [mg/l] **	407	3.54 (1.51-11.94)	6.67 (2.50-13.63)	1.003	0.998-1.009	0.260	ns	ns	ns
NPI, total score ***	352	3.38 ± 6.94	6.74 ± 11.67	**1.044**	**1.014-1.074**	**0.003**	ns	ns	ns
- agitation/aggression ***	352	1.24 ± 3.40	2.48 ± 6.61	**1.055**	**1.003-1.110**	**0.037**	ns	ns	ns
- mood ***	352	1.21 ± 2.69	2.91 ± 5.22	**1.121**	**1.054-1.192**	**<0.001**	**1.149**	**1.062-1.244**	**0.001**
- frontal ***	352	0.71 ± 1.74	1.85 ± 4.20	**1.154**	**1.056-1.260**	**0.002**	ns	ns	ns
- psychosis ***	352	0.16 ± 1.47	0.46 ± 2.08	1.099	0.961-1.258	0.169	ns	ns	ns
Pre-hospital IQCODE **	355	78 (78-79)	78 (78-84)	**1.057**	**1.020-1.095**	**0.002**	ns	ns	ns
Delirium in hospital *	421	21/289 (7.27%)	40/132 (30.30%)	**5.549**	**3.110-9.899**	**<0.001**	**4.828**	**2.225-10.477**	**<0.001**
Delirium type									
- hyperactive *	421	3/289 (1.04%)	11/132 (8.33%)	**8,667**	**2.376-31.617**	**0.001**	ns	ns	ns
- hypoactive *	421	10/289 (3.46%)	15/132 (11.36%)	**3.577**	**1.562-8.193**	**0.003**	ns	ns	ns
- mixed *	421	5/289 (1.73%)	10/132 (7.58%)	**4.656**	**1.559-13.907**	**0.006**	ns	ns	ns
Delirium length [days] **	61	3 (1-5)	3 (1-6)	1.009	0.795-1.281	0.941	ns	ns	ns

* n (%); ** median (IQR); *** mean ± SD; AES – Apathy Evaluation Scale; BMI – body mass index; TOAST – Trial of Org 10172 in Acute Stroke Treatment; rt-Pa – recombinant tissue plasminogen activator; PCI – percutaneous coronary interventions; CABG – coronary artery bypass graft; TIA – transient ischemic attack; CIRS – Cumulative Illness Rating Scale; NIHSS – National Institutes of Health Stroke Scale; mRS – Modified Rankin Scale; CRP – C-reactive protein; NPI – Neuropsychiatric Inventory; IQCODE - Informant Questionnaire on Cognitive Decline in the Elderly; ns – not significant

Table S9. Predictors of post-stroke apathy (AES score ≥ 37) at 3-month follow-up in univariate and multivariate logistic regression models

				Univariate logistic regression model			Multivariate logistic regression model		
**Variable**	**Data**	**No apathy**	**Apathy**	**OR**	**95%CI**	**P-value**	**OR**	**95%CI**	**P-value**
Male gender *	337	119/220 (54.09%)	54/117 (46.15%)	0.727	0.464-1.141	0.166	ns	ns	ns
Age [years] **	337	65 (58-75.5)	74 (64-81)	**1.046**	**1.026-1.068**	**<0.001**	ns	ns	ns
BMI [kg/m^2] **	330	25.53 (23.44-29.30)	27.34 (23.88-30.19)	**1.083**	**1.030-1.139**	**0.002**	**1.141**	**1.061-1.228**	**<0.001**
Higher education *	331	50/217 (23.04%)	14/114 (12.28%)	**0.468**	**0.246-0.889**	**0.020**	ns	ns	ns
Education length [years] **	326	11 (10-14)	11 (8-12)	**0.890**	**0.826-0.959**	**0.002**	ns	ns	ns
Hemorrhagic stroke *	337	8/220 (3.64%)	6/117 (5.13%)	1.432	0.485-4.231	0.516	ns	ns	ns
TOAST classification									
- 1 *	297	16/189 (8.47%)	9/108 (8.33%)	0.983	0.419-2.307	0.969	ns	ns	ns
- 2 *	297	9/189 (4.76%)	6/108 (5.56%)	1.176	0.407-3.400	0.764	ns	ns	ns
- 3 *	297	53/189 (28.04%)	46/108 (42.59%)	**1.904**	**1.159-3.127**	**0.011**	ns	ns	ns
- 4 *	297	109/189 (57.67%)	46/108 (42.59%)	**0.545**	**0.338-0.878**	**0.013**	ns	ns	ns
- 5 *	297	2/189 (1.06%)	1/108 (0.93%)	0.874	0.078-9.751	0.913	ns	ns	ns
Side of stroke									
- right hemisphere *	337	78/220 (35.45%)	50/117 (42.74%)	1.359	0.859-2.149	0.191	ns	ns	ns
- left hemisphere *	337	105/220 (47.73%)	55/117 (47.01%)	0.972	0.620-1.523	0.900	ns	ns	ns
- posterior part *	337	34/220 (15.45%)	11/117 (9.40%)	0.568	0.276-1.167	0.124	ns	ns	ns
- multiple locations *	337	3/220 (1.36%)	1/117 (0.85%)	0.624	0.064-6.062	0.684	ns	ns	ns
rt-Pa treatment *	337	52/220 (23.64%)	34/117 (29.06%)	1.323	0.798-2.195	0.277	ns	ns	ns
Thrombectomy *	337	12/220 (5.45%)	7/117 (5.98%)	1.103	0.422-2.882	0.841	ns	ns	ns
Medical history									
- hypertension *	337	144/220 (65.45%)	85/117 (72.65%)	1.402	0.857-2.294	0.179	ns	ns	ns
- diabetes *	337	48/220 (21.82%)	39/117 (33.33%)	**1.792**	**1.087-2.954**	**0.022**	ns	ns	ns
- atrial fibrillation *	337	28/220 (12.73%)	31/117 (26.50%)	**2.472**	**1.397-4.374**	**0.002**	ns	ns	ns
- myocardial infraction *	337	25/220 (11.36%)	20/117 (17.09%)	1.608	0.851-3.039	0.143	ns	ns	ns
- PCI or CABG *	337	18/220 (8.18%)	7/117 (5.98%)	0.714	0.289-1.763	0.465	ns	ns	ns
- smoking – ever *	336	110/219 (50.23%)	65/117 (55.56%)	1.239	0.789-1.944	0.352	ns	ns	ns
- smoking – current *	336	64/219 (29.22%)	29/117 (24.79%)	0.798	0.479-1.330	0.387	ns	ns	ns
- previous stroke or TIA *	337	32/220 (14.55%)	28/117 (23.93%)	**1.848**	**1.049-3.257**	**0.034**	ns	ns	ns
CIRS, total score **	337	7 (4-10)	10 (7-13)	**1.164**	**1.101-1.230**	**<0.001**	**1.125**	**1.036-1.223**	**0.005**
Medicines									
- anticholinergic risk scale ***	304	0.02 ± 0.28	0.24 ± 0.97	**2.055**	**1.052-4.014**	**0.035**	ns	ns	ns
- antidepressants *	277	4/183 (2.19%)	2/94 (2.13%)	0.973	0.175-5.411	0.975	ns	ns	ns
- neuroleptics *	277	2/183 (1.09%)	0/94 (0%)	-	-	-	-	-	-
- benzodiazepines *	276	4/182 (2.20%)	3/94 (3.19%)	1.467	0.321-6.695	0.621	ns	ns	ns
Pneumonia *	337	9/220 (4.09%)	12/117 (10.26%)	**2.679**	**1.094-6.560**	**0.031**	ns	ns	ns
Urinary tract infections *	326	41/214 (19.16%)	40/112 (35.71%)	**2.344**	**1.401-3.924**	**0.001**	ns	ns	ns
Hospital stay [days] **	337	9 (8-10)	9 (8-11)	**1.082**	**1.014-1.155**	**0.018**	ns	ns	ns
Aphasia in hospital *	337	49/220 (22.27%)	39/117 (33.33%)	**1.745**	**1.060-2.873**	**0.029**	ns	ns	ns
Neglect in hospital *	337	11/220 (5.00%)	11/117 (9.40%)	1.972	0.828-4.696	0.125	ns	ns	ns
Vision deficits in hospital *	337	39/220 (17.73%)	41/117 (35.04%)	**2.504**	**1.498-4.185**	**<0.001**	ns	ns	ns

NIHSS at admission **	337	3 (2-6.5)	6 (3-11)	**1.079**	**1.036-1.123**	**<0.001**	ns	ns	ns
Pre-hospital mRS **	337	0 (0-0)	0 (0-1)	**1.794**	**1.332-2.416**	**<0.001**	**1.878**	**1.092-3.229**	**0.023**
CRP in hospital [mg/l] **	326	3.54 (1.44-9.00)	6.34 (2.42-19.36)	**1.007**	**1.000-1.014**	**0.038**	ns	ns	ns
NPI, total score ***	277	3.68 ± 6.79	4.90 ± 9.15	1.020	0.988-1.053	0.218	ns	ns	ns
- agitation/aggression ***	277	1.39 ± 3.75	1.55 ± 4.18	1.010	0.949-1.075	0.747	ns	ns	ns
- mood ***	277	1.65 ± 4.08	1.74 ± 3.57	1.006	0.945-1.072	0.849	ns	ns	ns
- frontal ***	277	0.92 ± 2.31	1.01 ± 2.36	1.016	0.914-1.129	0.768	ns	ns	ns
- psychosis ***	277	0.09 ± 0.58	0.24 ± 1.18	1.231	0.907-1.670	0.183	ns	ns	ns
Pre-hospital IQCODE **	280	78 (78-78)	78 (78-82)	**1.111**	**1.046-1.179**	**<0.001**	**1.090**	**1.013-1.172**	**0.021**
Delirium in hospital *	337	15/220 (6.82%)	28/117 (23.93%)	**4.300**	**2.190-8.442**	**<0.001**	**3.841**	**1.315-11.216**	**0.014**
Delirium type									
- hyperactive *	337	3/220 (1.36%)	6/117 (5.13%)	3.910	0.960-15.930	0.057	ns	ns	ns
- hypoactive *	337	5/220 (2.27%)	11/117 (9.40%)	**4.462**	**1.152-13.172**	**0.007**	ns	ns	ns
- mixed *	337	3/220 (1.36%)	8/117 (6.84%)	**5.309**	**1.381-20.412**	**0.015**	ns	ns	ns
Delirium length [days] **	43	1 (1-6)	3 (1-6)	1.060	0.810-1.388	0.672	ns	ns	ns

* n (%); ** median (IQR); *** mean ± SD; AES – Apathy Evaluation Scale; BMI – body mass index; TOAST – Trial of Org 10172 in Acute Stroke Treatment; rt-Pa – recombinant tissue plasminogen activator; PCI – percutaneous coronary interventions; CABG – coronary artery bypass graft; TIA – transient ischemic attack; CIRS – Cumulative Illness Rating Scale; NIHSS – National Institutes of Health Stroke Scale; mRS – Modified Rankin Scale; CRP – C-reactive protein; NPI – Neuropsychiatric Inventory; IQCODE - Informant Questionnaire on Cognitive Decline in the Elderly; ns – not significant

Table S10. Predictors of post-stroke apathy (AES score ≥ 37) at 12-month follow-up in univariate and multivariate logistic regression models

				Univariate logistic regression model			Multivariate logistic regression model		
**Variable**	**Data**	**No apathy**	**Apathy**	**OR**	**95%CI**	**P-value**	**OR**	**95%CI**	**P-value**
Male gender *	231	94/174 (54.02%)	29/57 (50.88%)	0.881	0.484-1.604	0.680	ns	ns	ns
Age [years] **	231	65 (56-72)	73 (64-79)	**1.062**	**1.031-1.094**	**<0.001**	**1.047**	**1.010-1.086**	**0.012**
BMI [kg/m²] **	228	26.09 (24.09-30.07)	24.69 (22.86-27.74)	**0.927**	**0.864-0.995**	**0.036**	ns	ns	ns
Higher education *	229	43/172 (25.00%)	9.57 (15.79%)	0.563	0.255-1.241	0.154	ns	ns	ns
Education length [years] **	226	12 (10-15)	11 (10-12.5)	**0.875**	**0.789-0.970**	**0.011**	ns	ns	ns
Hemorrhagic stroke *	231	11/174 (6.32%)	1/57 (1.75%)	0.265	0.033-2.096	0.208	ns	ns	ns
TOAST classification									
- 1 *	195	16/146 (10.96%)	5/49 (10.20%)	0.923	0.320-2.67	0.883	ns	ns	ns
- 2 *	195	4/146 (2.74%)	2/49 (4.08%)	1.511	0.268-8.514	0.640	ns	ns	ns
- 3 *	195	41/146 (28.08%)	15/49 (30.61%)	1.130	0.557-2.290	0.735	ns	ns	ns
- 4 *	195	84/146 (57.53%)	26/49 (53.06%)	0.834	0.436-1.598	0.585	ns	ns	ns
- 5 *	195	1/146 (0.68%)	1/49 (2.04%)	3.021	0.185-49.232	0.438	ns	ns	ns
Side of stroke									
- right hemisphere *	231	58/174 (33.33%)	28/57 (49.12%)	**1.931**	**1.052-3.545**	**0.034**	**2.196**	**1.022-4.719**	**0.044**
- left hemisphere *	231	81/174 (46.55%)	27/57 (47.37%)	1.033	0.568-1.881	0.915	ns	ns	ns
- posterior part *	231	32/174 (18.39%)	2/57 (3.51%)	**0.161**	**0.037-0.696**	**0.015**	ns	ns	ns
- multiple locations *	231	3/174 (1.72%)	0/57 (0%)	-	-	-	-	-	-
rt-Pa treatment *	231	37/174 (21.26%)	12/57 (21.05%)	0.987	0.474-2.055	0.973	ns	ns	ns
Thrombectomy *	231	9/174 (5.17%)	4/57 (7.02%)	1.384	0.409-4.676	0.601	ns	ns	ns
Medical history									
- hypertension *	231	120/174 (68.97%)	49/57 (85.96%)	**2.756**	**1.222-6.217**	**0.015**	ns	ns	ns
- diabetes *	231	40/174 (22.99%)	20/57 (35.09%)	1.811	0.947-3.464	0.073	ns	ns	ns
- atrial fibrillation *	231	19/174 (10.92%)	15/57 (26.32%)	**2.914**	**1.365-6.217**	**0.006**	ns	ns	ns
- myocardial infraction *	231	24/174 (13.79%)	10/57 (17.54%)	1.330	0.593-2.981	0.489	ns	ns	ns
- PCI or CABG *	231	17/174 (9.77%)	4/57 (7.02%)	0.697	0.225-2.164	0.532	ns	ns	ns
- smoking – ever *	230	88/174 (50.57%)	27/56 (48.21%)	0.910	0.498-1.662	0.759	ns	ns	ns
- smoking – current *	230	42/174 (24.14%)	15/56 (26.79%)	1.150	0.579-2.283	0.690	ns	ns	ns
- previous stroke or TIA *	230	23/173 (13.29%)	13/57 (22.81%)	1.927	0.902-4.114	0.090	ns	ns	ns
CIRS, total score **	231	7 (4-10)	9 (6-13)	**1.137**	**1.057-1.222**	**<0.001**	ns	ns	ns
Medicines									
- anticholinergic risk scale ***	213	0.00 ± 0.00	0.17 ± 0.72	-	-	-	-	-	-
- antidepressants *	190	0/144 (0%)	3/46 (6.52%)	-	-	-	-	-	-
- neuroleptics *	190	1/144 (0.69%)	0/46 (0%)	-	-	-	-	-	-
- benzodiazepines *	190	1/144 (0.69%)	1/46 (2.17%)	3.178	0.195-51.843	0.417	ns	ns	ns
Pneumonia *	231	6/174 (3.45%)	3/57 (5.26%)	1.556	0.376-6.432	0.542	ns	ns	ns
Urinary tract infections *	224	30/170 (17.65%)	10/54 (18.52%)	1.061	0.480-2.341	0.884	ns	ns	ns
Hospital stay [days] **	231	9 (8-11)	9 (8-10)	0.986	0.985-1.087	0.780	ns	ns	ns
Aphasia in hospital *	231	43/174 (24.71%)	17/57 (29.82%)	1.295	0.667-2.515	0.446	ns	ns	ns
Neglect in hospital *	231	9/174 (5.17%)	8/57 (14.04%)	2.993	1.096-8.172	0.032	ns	ns	ns
Vision deficits in hospital *	231	34/174 (19.54%)	17/57 (29.82%)	1.750	0.887-3.454	0.107	ns	ns	ns

NIHSS at admission **	231	3 (1-6)	5 (2-10)	1.042	0.989-1.097	0.123	ns	ns	ns
Pre-hospital mRS **	231	0 (0-0)	0 (0-1)	**2.141**	**1.361-3.366**	**0.001**	**1.791**	**1.033-3.104**	**0.038**
CRP in hospital [mg/l] **	224	2.77 (1.37-10.19)	4.22 (1.68-14.35)	1.002	0.994-1.011	0.632	ns	ns	ns
NPI, total score ***	190	2.56 ± 5.39	5.46 ± 8.22	**1.065**	**1.015-1.118**	**0.011**	**1.055**	**1.001-1.113**	**0.046**
- agitation/aggression ***	190	0.95 ± 3.17	2.00 ± 4.53	1.072	0.985-1.168	0.107	ns	ns	ns
- mood ***	190	1.26 ± 3.15	2.07 ± 4.61	1.058	0.972-1.150	0.192	ns	ns	ns
- frontal ***	190	0.76 ± 2.18	1.17 ± 2.52	1.075	0.942-1.226	0.286	ns	ns	ns
- psychosis ***	190	0.08 ± 0.59	0.17 ± 0.90	1.184	0.770-1.821	0.442	ns	ns	ns
Pre-hospital IQCODE **	191	78 (78-78)	78 (78-81)	**1.136**	**1.038-1.243**	**0.006**	ns	ns	ns
Delirium in hospital *	231	9/174 (5.17%)	13/57 (22.81%)	**5.417**	**2.175-13.493**	**<0.001**	**4.951**	**1.685-14.547**	**0.004**
Delirium type									
- hyperactive *	231	4/174 (2.30%)	3/57 (5.26%)	2.361	0.512-10.882	0.270	ns	ns	ns
- hypoactive *	231	3/174 (1.72%)	4/57 (7.02%)	4.302	0.933-19.834	0.061	ns	ns	ns
- mixed *	231	0/174 (0%)	2/57 (3.51%)	-	-	-	-	-	-
Delirium length [days] **	22	2 (1-6)	3 (1-6)	1.024	0.720-1.455	0.896	ns	ns	ns

* n (%); ** median (IQR); *** mean ± SD; AES – Apathy Evaluation Scale; BMI – body mass index; TOAST – Trial of Org 10172 in Acute Stroke Treatment; rt-Pa – recombinant tissue plasminogen activator; PCI – percutaneous coronary interventions; CABG – coronary artery bypass graft; TIA – transient ischemic attack; CIRS – Cumulative Illness Rating Scale; NIHSS – National Institutes of Health Stroke Scale; mRS – Modified Rankin Scale; CRP – C-reactive protein; NPI – Neuropsychiatric Inventory; IQCODE - Informant Questionnaire on Cognitive Decline in the Elderly; ns – not significant

Table S11. Predictors of post-stroke anxiety (STAI-S sten score ≥ 7) at 3-month follow-up in univariate and multivariate logistic regression models

				Univariate logistic regression model			Multivariate logistic regression model		
**Variable**	**Data**	**No anxiety**	**Anxiety**	**OR**	**95%CI**	**P-value**	**OR**	**95%CI**	**P-value**
Male gender *	345	138/252 (54.76%)	45/93 (48.39%)	0.774	0.481-1.247	0.293	ns	ns	ns
Age [years] **	345	68 (59-78)	69 (63-78)	1.009	0.990-1.028	0.362	ns	ns	ns
BMI [kg/m^2] **	338	25.75 (23.44-29.35)	27.17 (23.88-31.01)	**1.061**	**1.008-1.116**	**0.024**	**1.087**	**1.019-1.159**	**0.011**
Higher education *	339	52/247 (21.05%)	14/92 (15.22%)	0.673	0.353-1.284	0.230	ns	ns	ns
Education length [years] **	332	11 (10-13)	11 (9-13)	0.956	0.886-1.030	0.239	ns	ns	ns
Hemorrhagic stroke *	345	13/252 (5.16%)	3/93 (3.23%)	0.613	0.171-2.201	0.453	ns	ns	ns
TOAST classification									
- 1 *	305	22/220 (10.00%)	9/85 (10.59%)	1.066	0.470-2.418	0.879	ns	ns	ns
- 2 *	305	13/220 (5.91%)	6/85 (7.06%)	1.209	0.444-3.292	0.710	ns	ns	ns
- 3 *	305	63/220 (28.64%)	34/85 (40.00%)	1.661	0.985-2.803	0.057	ns	ns	ns
- 4 *	305	121/220 (55.00%)	34/85 (40.00%)	**0.545**	**0.328-0.907**	**0.020**	ns	ns	ns
- 5 *	305	1/220 (0.45%)	2/85 (2.35%)	5.277	0.472-58-976	0.177	ns	ns	ns
Side of stroke									
- right hemisphere *	345	89/252 (35.32%)	39/93 (41.94%)	1.323	0.813-2.151	0.260	ns	ns	ns
- left hemisphere *	345	124/252 (49.21%)	46/93 (49.46%)	1.010	0.628-1.626	0.966	ns	ns	ns
- posterior part *	345	36/252 (14.29%)	7/93 (7.53%)	0.488	0.209-1.140	0.097	ns	ns	ns
- multiple locations *	345	3/252 (1.19%)	1/93 (1.08%)	0.902	0.093-8.783	0.929	ns	ns	ns
rt-Pa treatment *	345	56/252 (22.22%)	28/93 (30.11%)	1.508	0.884-2.570	0.131	ns	ns	ns
Thrombectomy *	345	11/252 (4.37%)	8/93 (8.60%)	2.062	0.803-5.298	0.133	ns	ns	ns
Medical history									
- hypertension *	345	172/252 (68.25%)	68/93 (73.12%)	1.265	0.745-2.149	0.384	ns	ns	ns
- diabetes *	345	63/252 (25.00%)	28/93 (30.11%)	1.292	0.763-2.189	0.340	ns	ns	ns
- atrial fibrillation *	345	39/252 (15.48%)	19/93 (20.43%)	1.402	0.763-2.578	0.276	ns	ns	ns
- myocardial infraction *	345	33/252 (13.10%)	16/93 (17.20%)	1.379	0.719-2.644	0.333	ns	ns	ns
- PCI or CABG *	345	22/252 (8.73%)	6/93 (6.45%)	0.721	0.283-1.838	0.493	ns	ns	ns
- smoking – ever *	344	126/251 (50.20%)	57/93 (61.29%)	1.571	0.967-2.551	0.068	ns	ns	ns
- smoking – current *	344	67/251 (26.69%)	30/93 (32.26%)	1.308	0.780-2.193	0.309	ns	ns	ns
- previous stroke or TIA *	345	38/252 (15.08%)	26/93 (27.96%)	**2.185**	**1.237-3.862**	**0.007**	**2.385**	**1.127-5.047**	**0.023**
CIRS, total score **	345	7 (5-11)	10 (7-13)	**1.129**	**1.069-1.192**	**<0.001**	**1.086**	**1.010-1.168**	**0.026**
Medicines									
- anticholinergic risk scale ***	309	0.06 ± 0.40	0.12 ± 0.71	1.244	0.790-1.961	0.346	ns	ns	ns
- antidepressants *	287	0/211 (0%)	2/76 (2.63%)	-	-	-			
- neuroleptics *	287	2.211 (0.95%)	0/76 (0%)	-	-	-			
- benzodiazepines *	286	4/210 (1.90%)	1/76 (1.32%)	0.687	0.076-6.242	0.739	ns	ns	ns
Pneumonia *	345	13/252 (5.16%)	10/93 (10.75%)	2.215	0.936-5.242	0.070	ns	ns	ns
Urinary tract infections *	333	53/243 (21.81%)	36/90 (40.00%)	**2.390**	**1.421-4.021**	**0.001**	**2.086**	**1.080-4.026**	**0.029**
Hospital stay [days] **	345	9 (8-10)	9 (8-11)	1.043	0.980-1.110	0.188	ns	ns	ns
Aphasia in hospital *	345	66/252 (26.19%)	26/93 (27.96%)	1.094	0.642-1.863	0.742	ns	ns	ns
Neglect in hospital *	345	16/252 (6.35%)	8/93 (8.60%)	1.388	0.573-3.361	0.467	ns	ns	ns
Vision deficits in hospital *	345	60/252 (23.81%)	28/93 (30.11%)	1.378	0.812-2.341	0.235	ns	ns	ns

NIHSS at admission **	345	4 (2-7)	5 (3-10)	1.040	0.998-1.084	0.064	ns	ns	ns
Pre-hospital mRS **	345	0 (0-0)	0 (0-0)	**1.330**	**1.026-1.723**	**0.031**	ns	ns	ns
CRP in hospital [mg/l] **	335	3.85 (1.59-10.66)	6.60 (2.41-18.10)	1.004	0.997-1.010	0.253	ns	ns	ns
NPI, total score ***	287	3.71 ± 8.09	6.92 ± 9.34	**1.040**	**1.010-1.071**	**0.009**	ns	ns	ns
- agitation/aggression ***	287	1.48 ± 4.43	1.76 ± 3.14	1.016	0.957-1.079	0.607	ns	ns	ns
- mood ***	287	1.16 ± 2.73	1.78 ± 3.12	1.071	0.984-1.116	0.112	ns	ns	ns
- frontal ***	287	1.29 ± 3.46	2.00 ± 3.46	1.055	0.984-1.131	0.135	ns	ns	ns
- psychosis ***	287	0.13 ± 0.83	0.47 ± 2.32	1.171	0.959-1.429	0.121	ns	ns	ns
Pre-hospital IQCODE **	290	78 (78-79.5)	78 (78-82)	1.028	0.995-1.063	0.095	ns	ns	ns
Delirium in hospital *	345	26/252 (10.32%)	25/93 (26.88%)	**3.196**	**1.732-5.895**	**<0.001**	**2.831**	**1.254-6.391**	**0.012**
Delirium type									
- hyperactive *	345	4/252 (1.59%)	4/93 (4.30%)	2.787	0.682-11.378	0.153	ns	ns	ns
- hypoactive *	345	11/252 (4.37%)	7/93 (7.53%)	1.783	0.670-4.747	0.247	ns	ns	ns
- mixed *	345	6/252 (2.38%)	10/93 (10.75%)	**4.940**	**1.742-14.007**	**0.003**	ns	ns	ns
Delirium length [days] **	51	1.5 (1-5)	3 (1-6)	1.136	0.898-1.438	0.288	ns	ns	ns

* n (%); ** median (IQR); *** mean ± SD; STAI-S – State-Trait Anxiety Inventory, state scale; BMI – body mass index; TOAST – Trial of Org 10172 in Acute Stroke Treatment; rt-Pa – recombinant tissue plasminogen activator; PCI – percutaneous coronary interventions; CABG – coronary artery bypass graft; TIA – transient ischemic attack; CIRS – Cumulative Illness Rating Scale; NIHSS – National Institutes of Health Stroke Scale; mRS – Modified Rankin Scale; CRP – C-reactive protein; NPI – Neuropsychiatric Inventory; IQCODE - Informant Questionnaire on Cognitive Decline in the Elderly; ns – not significant

Table S12. Predictors of post-stroke aggression (BDHI 'Aggression' sten score ≥ 7) in hospital in univariate and multivariate logistic regression models

				Univariate logistic regression model			Multivariate logistic regression model		
**Variable**	**Data**	**No aggression**	**Aggression**	**OR**	**95%CI**	**P-value**	**OR**	**95%CI**	**P-value**
Male gender *	441	200/403 (49.63%)	22/38 (57.89%)	1.396	0.712-2.735	0.332	ns	ns	ns
Age [years] **	441	68 (60-79)	78 (69-84)	0.995	0.971-1.020	0.689	ns	ns	ns
BMI [kg/m2] **	432	27.16 (23.88-30.47)	26.45 (24.09-29.74)	1.021	0.955-1.091	0.544	ns	ns	ns
Higher education *	436	84/399 (21.05%)	4/37 (10.81%)	0.455	0.157-1.319	0.147	ns	ns	ns
Education length [years] **	435	11 (10-13)	10 (8-12)	0.954	0.858-1.061	0.389	ns	ns	ns
Hemorrhagic stroke *	441	22/403 (5.46%)	4/38 (10.53%)	2.037	0.664-6.255	0.214	ns	ns	ns
TOAST classification									
- 1 *	383	39/349 (11.17%)	7/34 (20.59%)	2.061	0.842-5.046	0.114	ns	ns	ns
- 2 *	383	21/349 (6.02%)	2/34 (5.88%)	0.976	0.219-4.354	0.975	ns	ns	ns
- 3 *	383	101/349 (28.94%)	12/34 (35.29%)	1.339	0.639-2.808	0.439	ns	ns	ns
- 4 *	383	184/349 (52.72%)	12/34 (35.29%)	0.489	0.235-1.019	0.056	ns	ns	ns
- 5 *	383	4/349 (1.15%)	1/34 (2.94%)	2.614	0.284-24.071	0.396	ns	ns	ns
Side of stroke									
- right hemisphere *	441	172/403 (42.68%)	19/38 (50.00%)	1.343	0.690-2.614	0.385	ns	ns	ns
- left hemisphere *	441	168/403 (41.69%)	16/38 (42.11%)	1.017	0.519-1.996	0.960	ns	ns	ns
- posterior part *	441	53/403 (13.15%)	2/38 (5.26%)	0.367	0.086-1.569	0.176	ns	ns	ns
- multiple locations *	441	10/403 (2.48%)	1/38 (2.63%)	1.062	0.132-8.529	0.955	ns	ns	ns
rt-Pa treatment *	441	100/403 (24.81%)	5/38 (13.16%)	0.459	0.174-1.208	0.115	ns	ns	ns
Thrombectomy *	441	18/403 (4.47%)	1/38 (2.63%)	0.578	0.075-4.454	0.599	ns	ns	ns
Medical history									
- hypertension *	441	277/403 (68.73%)	26/38 (68.42%)	0.986	0.482-2.016	0.968	ns	ns	ns
- diabetes *	441	104/403 (25.81%)	12/38 (31.58%)	1.327	0.646-2.725	0.441	ns	ns	ns
- atrial fibrillation *	441	67/403 (16.63%)	10/38 (26.32%)	1.791	0.831-3.861	0.137	ns	ns	ns
- myocardial infraction *	441	55/403 (14.65%)	4/38 (10.53%)	0.744	0.254-2.180	0.590	ns	ns	ns
- PCI or CABG *	441	33/403 (8.19%)	6/38 (15.79%)	2.102	0.820-5.392	0.122	ns	ns	ns
- smoking – ever *	440	201/402 (50.00%)	27/38 (71.05%)	**2.455**	**1.185-5.082**	**0.016**	**3.088**	**1.333-7.154**	**0.009**
- smoking – current *	440	111/402 (27.61%)	16/38 (42.11%)	1.907	0.966-3.764	0.063	ns	ns	ns
- previous stroke or TIA *	439	71/401 (17.71%)	5/38 (13.16%)	0.704	0.266-1.867	0.481	ns	ns	ns
CIRS, total score **	441	8 (5-12)	10 (7-14)	1.027	0.958-1.102	0.451	ns	ns	ns
Medicines									
- anticholinergic risk scale ***	401	0.05 ± 0.35	0.14 ± 0.76	1.656	0.971-2.825	0.064	ns	ns	ns
- antidepressants *	368	9/333 (2.70%)	1/35 (2.86%)	1.059	0.130-8.612	0.957	ns	ns	ns
- neuroleptics *	368	2/333 (0.60%)	0/35 (0%)	-	-	-	-	-	-
- benzodiazepines *	367	5/332 (1.51%)	0/35 (0%)	-	-	-	-	-	-
Pneumonia *	441	18/403 (4.47%)	4/38 (10.53%)	2.516	0.806-7.858	0.112	ns	ns	ns
Urinary tract infections *	426	110/392 (28.06%)	8/34 (23.53%)	0.789	0.347-1.795	0.572	ns	ns	ns
Hospital stay [days] **	441	9 (8-11)	11 (8-15)	1.070	0.999-1.146	0.055	ns	ns	ns
Aphasia in hospital *	441	76/403 (18.86%)	8/38 (21.05%)	1.147	0.506-2.602	0.742	ns	ns	ns
Neglect in hospital *	441	42/403 (10.42%)	7/38 (18.42%)	1.941	0.805-4.680	0.140	ns	ns	ns
Vision deficits in hospital *	441	107/403 (26.55%)	13/38 (34.21%)	1.439	0.710-2.913	0.313	ns	ns	ns

NIHSS at admission **	441	4 (2-8)	15 (7-20)	**1.070**	**1.013-1.132**	**0.016**	**1.141**	**1.060-1.227**	**<0.001**
Pre-hospital mRS **	441	0 (0-0)	0 (0-2)	1.043	0.752-1.446	0.800	ns	ns	ns
CRP in hospital [mg/l] **	427	4.41 (1.77-11.97)	13.46 (4.53-48.56)	1.002	0.994-1.010	0.645	ns	ns	ns
NPI, total score ***	368	4.72 ± 9.36	5.20 ± 9.46	1.005	0.971-1.040	0.775	ns	ns	ns
- agitation/aggression ***	368	1.05 ± 3.11	1.57 ± 3.37	1.041	0.955-1.134	0.362	ns	ns	ns
- mood ***	368	1.74 ± 4.01	2.54 ± 5.13	1.038	0.970-1.112	0.279	ns	ns	ns
- frontal ***	368	1.40 ± 4.05	1.89 ± 4.23	1.024	0.955-1.097	0.506	ns	ns	ns
- psychosis ***	368	0.32 ± 2.03	0.00 ± 0.00	-	-	-	-	-	-
Pre-hospital IQCODE **	371	78 (78-81)	78 (78-81)	0.992	0.941-1.045	0.756	ns	ns	ns
Delirium in hospital *	441	62/403 (15.38%)	11/38 (28.95%)	**2.241**	**1.057-4.751**	**0.035**	**3.391**	**1.486-7.739**	**0.004**
Delirium type									
- hyperactive *	441	12403 (2.98%)	2/38 (5.26%)	1.810	0.390-8.405	0.449	ns	ns	ns
- hypoactive *	441	28/403 (6.95%)	1/38 (2.63%)	0.362	0.048-2.737	0.325	ns	ns	ns
- mixed *	441	13/403 (3.23%)	8/38 (21.05%)	**8.000**	**3.076-20.807**	**<0.001**	ns	ns	ns
Delirium length [days] **	73	2.5 (1-6)	4 (3-7)	1.202	0.917-1.577	0.183	ns	ns	ns

* n (%); ** median (IQR); *** mean ± SD; BDHI – Buss-Durkee Hostility Inventory; 'Aggression' – assault, indirect hostility, irritability and verbal hostility; BMI – body mass index; TOAST – Trial of Org 10172 in Acute Stroke Treatment; rt-Pa – recombinant tissue plasminogen activator; PCI – percutaneous coronary interventions; CABG – coronary artery bypass graft; TIA – transient ischemic attack; CIRS – Cumulative Illness Rating Scale; NIHSS – National Institutes of Health Stroke Scale; mRS – Modified Rankin Scale; CRP – C-reactive protein; NPI – Neuropsychiatric Inventory;IQCODE - Informant Questionnaire on Cognitive Decline in the Elderly; ns – not significant

Table S13. Differences in baseline characteristics between lost to follow-up patients and retained patients at 3-month follow-up

Variable	Data	Lost to follow-up (43)	Retained patients (572)	P-value
Male gender *	615	19/43 (44.19%)	277/572 (48.43%)	0.591
Age [years] **	615	72 (65-84)	70 (61-79.5)	**0.033**
Higher education *	589	10/36 (27.78%)	99/553 (17.90%)	0.139
Education length [years] **	572	10 (8-16)	11 (9-13)	0.410
Hemorrhagic stroke *	615	4/43 (9.30%)	33/572 (5.77%)	0.544
rt-Pa treatment *	615	6/43 (13.95%)	146/572 (25.52%)	0.130
Thrombectomy *	615	0/43 (0%)	28/572 (4.90%)	0.269
Previous stroke or TIA *	613	9/43 (20.93%)	106/570 (18.60%)	0.861
CIRS, total score **	615	9 (5-13)	9 (5-12)	0.228
NIHSS at admission **	615	7 (1-16)	5 (2-11)	0.373
Pre-hospital mRS **	615	0 (0-2)	0 (0-0)	**0.031**
NPI, total score ***	509	9.86 ± 4.85	6.83 ± 12.08	0.242
Pre-hospital IQCODE **	512	79 (78-95)	78 (78-82)	**0.038**
Delirium in hospital *	615	11/43 (25.58%)	114/572 (19.93%)	0.374
Delirium type				
- hyperactive *	615	3/43 (6.98%)	21/572 (3.67%)	0.502
- hypoactive *	615	2/43 (4.65%)	43/572 (7.52%)	0.695
- mixed *	615	6/43 (13.95%)	40/572 (6.99%)	0.170
Delirium length [days] **	125	4 (2-7)	3 (2-7)	0.294

* n (%); ** median (IQR); *** mean ± SD; BMI – body mass index; rt-Pa – recombinant tissue plasminogen activator; TIA – transient ischemic attack; CIRS – Cumulative Illness Rating Scale; NIHSS – National Institutes of Health Stroke Scale; mRS – Modified Rankin Scale; NPI – Neuropsychiatric Inventory; IQCODE - Informant Questionnaire on Cognitive Decline in the Elderly

Table S14. Differences in baseline characteristics between lost to follow-up patients and retained patients at 12-month follow-up

Variable	Data	Lost to follow-up (78)	Retained patients (482)	P-value
Male gender *	560	37/78 (47.44%)	237 (49.17%)	0.776
Age [years] **	560	71.5 (64-83)	69 (60-79)	**0.013**
Higher education *	539	14/69 (20.29%)	92/470 (19.57%)	0.889
Education length [years] **	528	10 (8-14)	11 (10-13)	0.123
Hemorrhagic stroke *	560	7/78 (8.97%)	25/482 (5.19%)	0.283
rt-Pa treatment *	560	20/78 (25.64%)	116/482 (24.07%)	0.764
Thrombectomy *	560	2/78 (2.56%)	26/482 (5.39%)	0.433
Previous stroke or TIA *	558	15/78 (19.23%)	89/480 (18.54%)	0.885
CIRS, total score **	560	9 (5-13)	8 (5-12)	0.291
NIHSS at admission **	560	6 (3-14)	4 (2-9)	**0.043**
Pre-hospital mRS **	560	0 (0-1)	0 (0-0)	**0.040**
NPI, total score ***	460	9.21 ± 12.61	6.44 ± 12.19	0.111
Pre-hospital IQCODE **	463	78 (78-87)	78 (78-81)	0.131
Delirium in hospital *	560	23/78 (29.49%)	79/482 (16.39%)	**0.005**
Delirium type				
- hyperactive *	560	4/78 (5.13%)	16/482 (3.32%)	0.639
- hypoactive *	560	7/78 (8.97%)	30/482 (6.22%)	0.508
- mixed *	560	12/78 (15.38%)	25/482 (5.19%)	**<0.001**
Delirium length [days] **	102	3 (2-7)	3 (1-7)	0.870

* n (%); ** median (IQR); *** mean ± SD; BMI – body mass index; rt-Pa – recombinant tissue plasminogen activator; TIA – transient ischemic attack; CIRS – Cumulative Illness Rating Scale; NIHSS – National Institutes of Health Stroke Scale; mRS – Modified Rankin Scale; NPI – Neuropsychiatric Inventory; IQCODE - Informant Questionnaire on Cognitive Decline in the Elderly
